# Erythrocyte‐Inspired Functional Materials for Biomedical Applications

**DOI:** 10.1002/advs.202206150

**Published:** 2022-12-29

**Authors:** Zhiqiang Luo, Lingyu Sun, Feika Bian, Yu Wang, Yunru Yu, Zhuxiao Gu, Yuanjin Zhao

**Affiliations:** ^1^ Department of Rheumatology and Immunology Nanjing Drum Tower Hospital School of Biological Science and Medical Engineering Southeast University Nanjing 210096 China; ^2^ Oujiang Laboratory (Zhejiang Lab for Regenerative Medicine, Vision and Brain Health) Wenzhou Institute University of Chinese Academy of Sciences Wenzhou 325001 China

**Keywords:** biomaterial, erythrocyte, hydrogel, microfluidics, particle

## Abstract

Erythrocytes are the most abundant cells in the blood. As the results of long‐term natural selection, their specific biconcave discoid morphology and cellular composition are responsible for gaining excellent biological performance. Inspired by the intrinsic features of erythrocytes, various artificial biomaterials emerge and find broad prospects in biomedical applications such as therapeutic delivery, bioimaging, and tissue engineering. Here, a comprehensive review from the fabrication to the applications of erythrocyte‐inspired functional materials is given. After summarizing the biomaterials mimicking the biological functions of erythrocytes, the synthesis strategies of particles with erythrocyte‐inspired morphologies are presented. The emphasis is on practical biomedical applications of these bioinspired functional materials. The perspectives for the future possibilities of the advanced erythrocyte‐inspired biomaterials are also discussed. It is hoped that the summary of existing studies can inspire researchers to develop novel biomaterials; thus, accelerating the progress of these biomaterials toward clinical biomedical applications.

## Graphical abstract

A comprehensive review from the fabrication to the applications of erythrocyte‐inspired functional materials is given. Functionalized erythrocytes, erythrocyte membrane‐derived vesicles, hemoglobin‐based oxygen carrier, and morphology‐inspired particles with discoid shape, bowl shape, ring shape, and biconcave discoid shape are included. They are greatly developed in many fields, ranging from drug delivery and bioimaging to tissue engineering.

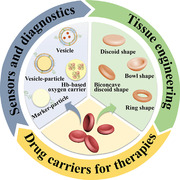

## 1 Introduction

Erythrocytes are the most abundant cells in the blood with optimized biological performances. They possess complex functionality attributing to unique biconcave discoid morphology, cellular components, and mechanical flexibility.^[^ ^1^, ^2^, ^3^ ^]^ As the results of long‐term natural selection, mature erythrocytes have no nucleus and organelles, which are mainly loaded with hemoglobin (Hb) inside and own large amounts of functional proteins residing on the membrane.^[^ ^4^ ^]^ The above cellular component characteristics play a critical role in realizing the multi‐functionalization of erythrocytes. To be specific, erythrocytes can circulate as cargo carriers to deliver oxygen and transport carbon dioxide for ≈120 days in the blood vessels before being eliminated due to senescence.^[^ ^5^ ^]^ In this process, the realization of oxygen delivery depends on the reversible oxygen binding capacity of Hb in erythrocytes;^[^ ^6^ ^]^ while the realization of prolonged circulation is mediated by a mass of membrane proteins to avoid immune clearance.^[^ ^7^ ^]^ In addition, the biconcave discoid morphology and mechanical flexibility of erythrocytes allow them to maintain a constant surface area even undergoing marked deformations, as well as provide a favorable surface area‐to‐volume ratio for gas exchange.^[^ ^8^, ^9^, ^10^ ^]^

Inspired by the above intrinsic features of erythrocytes, great progress has been achieved in the development of artificial materials to mimic the biological performance of erythrocytes. By referring to the functions of erythrocytes, some top–down technologies have leveraged erythrocytes and their membrane components as camouflage coating of drug carriers to escape from biological barriers.^[^ ^3^, ^11^ ^]^ Besides, the principle of Hb‐based oxygen delivery has also been successfully applied in the construction of artificial oxygen carriers (AOCs).^[^ ^12^ ^]^ By mimicking the biconcave discoid morphology feature of erythrocytes, synthetic non‐spherical particles with different shapes have been proposed. Recent studies reveal the active influence of non‐spherical morphology on cell activities and pharmacokinetics, which offer a novel mentality to optimize traditional spherical particles into non‐spherical ones for different biomedical applications, ranging from drug delivery and bioimaging to tissue engineering.^[^ ^13^, ^14^ ^]^ These biomaterials pave the avenue to combine the advantages of both biomaterial performance and biological entities featuring in biomedicine fields. In view of the extensive application potentials of the erythrocytes‐inspired functional materials in the biomedicine field, we believe that a timely and comprehensive review in this respect may facilitate the communication and progress of multiple disciplines, including material science and biomedicine.

In this paper, we focused on the cutting‐edge research on biomimetic materials that mimics the pivotal functional or structural features of erythrocytes. After summarizing the main strategies of erythrocytes refunctionalization, the erythrocyte membrane‐camouflaged drugs and nanoparticles (NPs), membrane proteins‐modified particles, and Hb‐based AOCs were introduced. Emphasis would be given to the fabrication methods and practical biomedical applications of non‐spherical particles mimicking erythrocyte morphology, including the ones with discoid shape, bowl shape, ring shape, and biconcave discoid shape. We hope the summary of existing research could inspire the development of novel biomaterials; thus, accelerating the progress of these biomaterials toward biomedical applications.

## 2 Biomaterials Mimicking the Biological Functions of Erythrocytes

### 2.1 Refunctionalization of Erythrocytes

**Figure 1:**
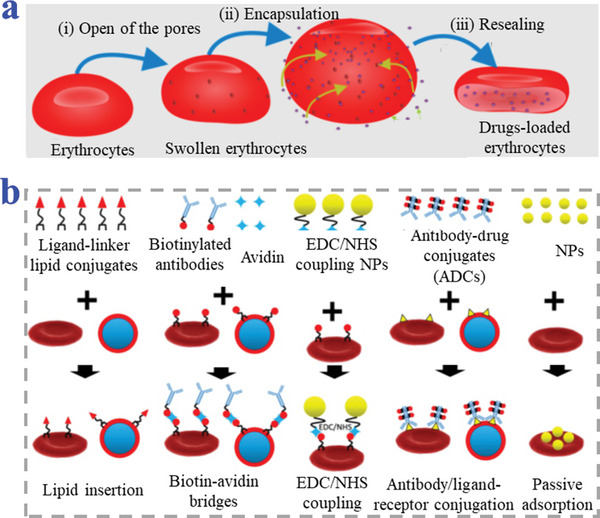
Strategies of erythrocytes refunctionalization. a) The internal loading strategy through hypotonic treatment. Reproduced with permission.^[^ ^16^ ^]^ Copyright 2018, The authors, Russian Open Medical Journal, Published by LLC Science and Innovations. b) The surface functionalization of natural erythrocytes. Reproduced under the terms of the CC‐BY license.^[^ ^15^ ^]^ Copyright 2019, The Authors, Published by Ivyspring International Publisher.

There are two main approaches to transform native erythrocytes into active carriers, namely surface loading and internal loading.^[^ ^15^ ^]^ To encapsulate drugs into erythrocytes, hypotonic treatment‐induced membrane lysis is one of the most widely applied strategies, such as hypotonic dialysis, hypotonic pre‐swelling, and hypotonic dilution. When treated in a hypotonic solution, erythrocyte membrane forms temporary pores and erythrocytes hemolyze under a net outward osmotic pressure, loading drugs into interiors through membrane pores. The opened pores subsequently can be resealed and the main properties restored (**Figure** **1**a).^[^ ^16^ ^]^ Electroporation is another method that works by forming temporary transmembrane pores, which relies on short, intense electrical charges. The membrane phospholipids reassemble due to reversible electric breakdown and membrane difference, forming hydrophilic pores to allow cargo entrance.^[^ ^17^ ^]^ There are other developed methods without physically disrupting cellular membranes to transport therapeutic compounds into erythrocytes. Drug molecules encapsulated with lipid vesicles could be directly fused with erythrocytes membranes to load drugs. Drugs induced erythrocytes endocytosis is another available strategy for internal loading.^[^ ^8^ ^]^ Newly, cell penetrating peptide (CPP) modified therapeutic can be transported into the erythrocytes after endocytosis. CPP can be covalently attached into proteins and drug carriers to cross cell membranes of all organ types.^[^ ^18^, ^19^, ^20^ ^]^ The feasibility of the above approaches supports the encapsulation of therapeutic proteins, liposomes, NPs, micelles, and so on.^[^ ^21^ ^]^

Further surface functionalization of natural erythrocytes adopting diverse techniques of either non‐covalent or covalent conjugation has been widely summarized, as shown in Figure 1b.^[^ ^15^, ^22^, ^23^ ^]^ Briefly, 1‐ethyl‐3‐(3‐dimethylaminopropyl) carbodiimide/N‐Hydroxysuccinimide (EDC/NHS) reaction could be applied to covalently conjugate molecules and NPs onto membrane. This direct chemical conjugation to erythrocytes membrane offers strong anchoring effect while either damaging erythrocytes membrane or consuming time. Non‐covalent conjugation strategies include lipid insertion, biotin–avidin bridges, and antibody–receptor conjugation. In these strategies, drugs and carriers need to be attached to lipids, peptides, antibodies/antibody fragments, and ligands with erythrocyte‐targeted capacity to be further transferred onto membrane. Non‐covalent conjugation methods are quite fast, easy, and would not disrupt the erythrocytes membrane. However, the conjugation may be less stable upon applied forces, causing the detaching of attached complex from erythrocytes. For instance, the complex would be pulled out from erythrocytes membrane by free proteins in the blood for their hydrophobic interactions. More recently, research reported that NPs could be attached directly to the surface of erythrocytes via either electrostatic, van der Waals, or hydrophobic interactions.^[^ ^24^, ^25^ ^]^ Inspired by this, erythrocytes hitchhiking, a passive adsorption method, is developed to facilitate NPs delivery through the transportation on erythrocytes membrane. The binding stability of this interaction is much weaker than the covalent conjugation. Thus, it is suitable to propose intelligent drug delivery systems responding to activation stimuli of cell/wall friction and high shear stress.^[^ ^15^ ^]^ In addition to therapeutic, other targeting ligands have also been coupled to the surface of erythrocyte membrane to enhance the target capability and/or promote the following cellular uptake. These ligands include angiopep‐2,^[^ ^26^ ^]^ folate,^[^ ^27^ ^]^ triphenylphosphonium,^[^ ^28^ ^]^ stroke homing peptide,^[^ ^29^ ^]^ and arginine‐glycine‐aspartate.^[^ ^30^ ^]^ Membrane fusion of erythrocytes with other cell membranes (e.g., cancer cells, immune cell, etc.) also endows extra targeting capacity for cell recognition.^[^ ^31^, ^32^ ^]^

In addition, genetic engineering has been developed as a common refunctionalization approach as well. Genetic engineering aims to make erythrocytes express therapeutic proteins (e.g., enzymes, antigens, receptor agonists, and combinations) in the cell or on the membrane to treat various diseases.^[^ ^32^ ^]^ Due to the lack of a nucleus in mature erythrocyte, this method focuses more on engineering of erythroid precursors (e. g. hematopoietic stem cells, progenitor cells) to make mature erythrocytes obtain target proteins.^[^ ^33^ ^]^ The final gene‐free erythrocytes would be controllable and safer than other cells to develop cell therapies, such as stem cell therapy and CAR‐T cell therapy.^[^ ^32^ ^]^

### 2.2 Biomaterial Mimicking the Stealth Properties

The unique transmembrane proteins residing on the lipid bilayer membrane make erythrocytes freely circulate in the cardiovascular system and organs with no attack and clearance actions from the immune system.^[^ ^34^ ^]^ It's vital for numerous biological activities, including cell adhesion, the reduction of the protein corona formation, and integrity maintenance. The most widely investigated transmembrane protein is CD47, which could recognize the signal‐regulatory protein *α* (SIRP*α*) on macrophage to give the signal of “do not eat me.”^[^ ^35^ ^]^ Inspired by the transmembrane proteins‐induced prolonged circulation of erythrocytes, scientists have adopted some top–down strategies to transform erythrocytes membrane as stealth coating materials of drugs or drug carriers to escape the immune clearance.^[^ ^3^ ^]^ Erythrocyte membrane‐camouflaged therapeutic is expected to achieve enhanced biocompatibility, get over biological barriers, locate at the target tissues in sufficient quantities, and reduce uptake of reticuloendothelial system/mononuclear phygocyte system (RES/MPS).^[^ ^36^ ^]^ Mature erythrocytes own no organelles and nucleus, facilitating the extraction and purification of erythrocyte membrane as well as decreasing safety risks. In addition, the erythrocyte membrane‐derived nanovesicles (EM‐NVs) are regarded to possess longer lifespans than the other cells vesicles.^[^ ^37^ ^]^ Furthermore, with recent advances in molecular and cellular biology of the membrane proteins on erythrocytes, the specific proteins are extracted and serve as self‐markers to be coupled on the carriers surface to achieve immune‐evasive functionalities.

#### 2.2.1 Erythrocytes Membrane‐Derived Nanovesicles

In a classical approach, erythrocytes are initially emptied to obtain the erythrocyte ghosts through hypotonic treatment, which typically includes erythrocyte lysis and membrane purification.^[^ ^38^ ^]^ During this process, a suitable balance must be established to harvest viable and stable structures of vesicle, avoiding the erythrocyte cleavage and the lysis medium.^[^ ^37^ ^]^ Repeated freeze‐thaw and sonication are also applied to obtain erythrocytes ghosts.^[^ ^31^, ^37^ ^]^ To translate the erythrocyte ghosts into nanosized EM‐NVs, sonication, extrusion, electrical breakdown, and combined methods are usually employed.^[^ ^11^, ^39^ ^]^ Collecting extracellular vesicles from culture media is also a useful way to get a small number of vesicles.^[^ ^40^ ^]^ It is worth mentioning that selective target capacity is a vital feature in applying EM‐NVs‐coated drugs as the therapy of diseases.^[^ ^32^ ^]^ The surface modification methods of erythrocytes could be applied to the functionalization of EM‐NVs as well (Figure 1b). Erythrocyte membrane owns the remarkable ability of fusing with other cells membranes, such as membranes of immune cell, platelet, cancer cell, stem cell, and even bacteria.^[^ ^31^ ^]^ Cell membrane coating technology enables the formation of hybrid nanovesicles of two or more types of cells by co‐culture to enhance the targetability of the carriers.^[^ ^41^, ^42^ ^]^ Currently, the hybrid cellular membranes are obtained through fusion of cells to membrane extraction or extraction of membranes to fusion.^[^ ^31^ ^]^ In the former method, the specific fusion process is normally induced by stimulation of electrofusion or polyethylene glycol. Notably, the protein components of obtained hybrid membrane may differ from the initial cells because this process induces a generation of new membrane proteins.^[^ ^43^ ^]^ Another approach is to extract membranes of different cells, respectively, and then to induce the membranes fusion by co‐extruding.^[^ ^7^ ^]^ These procedures are suitable to fusion with membranes from both eukaryotic cell and prokaryotic cell.^[^ ^44^ ^]^

#### 2.2.2 Erythrocytes Membrane‐Camouflaged Biomaterials

The development of EM‐NVs‐camouflaged NPs (EM‐NVs‐NPs) demonstrates a unique top–down method to propose biomimetic drug delivery systems. EM‐NVs‐NPs can behave like native erythrocytes while circulating in the blood, possessing prolonged circulation.^[^ ^45^ ^]^ Besides, those NPs keep the properties conferred on loading drugs, regulating release process, improving specificity, and protecting from degradation.^[^ ^46^, ^47^ ^]^ In this hybrid system, prepared NPs and EM‐NVs are fabricated, respectively. After mixing synthetic NPs with EM‐NVs, the reconstitution of EM‐NVs could appear on the surface of NPs through the induction of sonication, co‐incubation, mechanically induced physical extrusion, and microfluidic electroporation.^[^ ^48^, ^49^, ^50^ ^]^ The extrusion method was limited to coat various negatively charged NPs, including inorganic NPs, polymers, melanin, and quantum dots while the positively charged NPs would cause collapse or disorder of the lipid bilayer and further membrane aggregation due to the electrostatic interactions.^[^ ^47^ ^]^ In this hybrid system, the lipid bilayer coverage of core and *ζ* potential was close to the EM‐NVs.^[^ ^36^, ^51^ ^]^ The vesicle‐functionalized NPs without chemical conjugation still maintain functions and stability of the EM‐NVs in living organisms.^[^ ^45^ ^]^ Therefore, the strategy of EM‐NVs camouflage demonstrates a versatile design method of biomimetic delivery system for biomedical applications.

#### 2.2.3 Erythrocytes Self‐Markers Modified Biomaterials

The underlying mechanism that erythrocytes used to evade the immune system is the interactions between the surface glycoproteins and signal‐regulatory proteins of immune cells, thereby inhibiting phagocytosis.^[^ ^11^ ^]^ The translocation of membrane proteins to the surface of synthetic particles, serving as self‐markers, allows artificial carriers to share perfected functionalities of natural erythrocytes.^[^ ^52^ ^]^ The surface glycoproteins, especially the CD47, act as signaling molecules to inhibit phagocytes and macrophages.^[^ ^52^, ^53^ ^]^ In addition, decay‐accelerating factor (DAF) and complement receptor 1 on erythrocyte surfaces could escape the attack from the complement system.^[^ ^54^ ^]^ C8 binding protein and CD59 on the erythrocyte membrane could inhibit the assembly of the hemolytic pore‐forming membrane attack complex to manage the late‐stage complement cascade.^[^ ^55^, ^56^ ^]^ Other less noticed membrane proteins include homologous restriction protein and membrane cofactor protein. These findings inspire researchers to attach extracted self‐marker proteins as the surface decoration of the particles. Compared to the traditional polyethylene glycol (PEG) modification strategy, this biomimetic strategy has represented superiorities in prolonging circulation time, facilitating cell uptake, improving tumor accumulation, and tuning intracellular fate.^[^ ^11^, ^57^ ^]^ For instance, in the work by Tsai et al., phagocytosis was observed to be reduced by ≈50% in CD47‐coated NPs compared to the CD47‐free one.^[^ ^58^ ^]^ Rodriguez et al. found that the half‐life of the CD47‐NPs increased up to a fourfold than the control in the bloodstream, improving drug retention in the tumor.^[^ ^59^ ^]^

### 2.3 Artificial Erythrocyte

**Figure 2:**
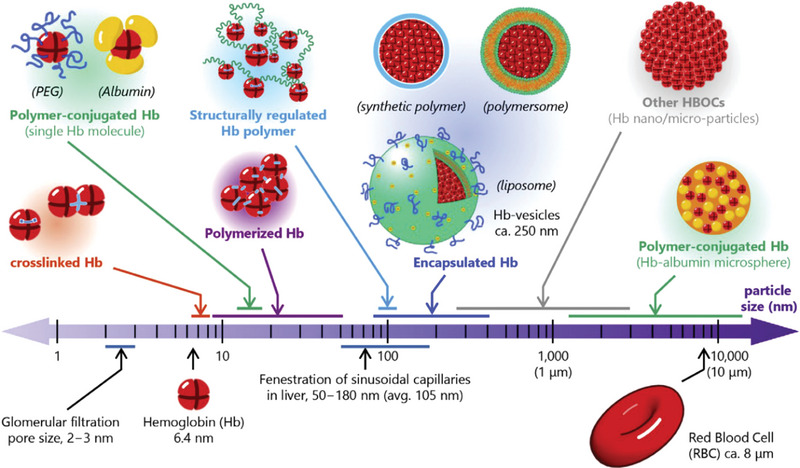
Various HBOCs from nanometer‐sized to micrometer‐sized. Reproduced with permission.^[^ ^12^ ^]^ Copyright 2021, Chinese Society of Particuology and Institute of Process Engineering, Chinese Academy of Sciences, Published by Elsevier

Sustaining life needs oxygen and diseases treatment requires additional oxygen input. The realization of oxygen delivery in vivo largely depends on the reversible oxygen binding capacity of Hb in erythrocyte, which is the most ubiquitous protein in the blood.^[^ ^60^ ^]^ The idea of AOCs is to supply oxygen needed by tissues and organs to deal with insufficient oxygen supply of erythrocytes. Inspired by the excellent oxygen carrying performance of Hb in erythrocytes, AOCs based on Hb are considered as the most promising oxygen carriers to mimic erythrocytes.^[^ ^61^ ^]^ However, cell‐free Hb would easily dissociate into dimers in physiological environment, causing serious adverse effects such as vasoconstriction and renal toxicity.^[^ ^62^ ^]^ Hb‐based oxygen carriers (HBOCs) are semi‐synthetic systems which utilize the excellent properties of natural Hb in O_2_ bonding and delivery (**Figure** **2**). They aim at avoiding or at least minimizing extravasation, dissociation, and rapid clearance of Hb by the kidneys.^[^ ^61^ ^]^ Early explorations of HBOCs primarily depended on non‐site‐specific modifications of the Hb such as intramolecular cross‐linking, polymerization, and conjugation with polymer. Cross‐linking of Hb has been developed by conjugation in subunits intramolecularly and two Hb molecules.^[^ ^12^ ^]^ Polymerized Hb was developed by using aldehydes (e.g., glutaraldehyde, formaldehyde) as fixatives. Besides, toxicity of Hb in polymer‐conjugated Hb was reduced when the conjugated polymers were non‐immunogenic and highly biocompatible such as dextran, albumin, and PEG. However, the stroma‐free Hb in those chemically synthetic HBOCs reserved some adverse effects of Hb molecularly, including hypertension, vasoconstriction, renal toxicity, and higher incidence of infarction.^[^ ^62^ ^]^ What's more, the extended exposure of these HBOCs in bloodstream directly exposes Hb to the self‐degradative processes and the rapid oxidation of HBOCs has also triggered other innate immune responses.^[^ ^63^ ^]^

Recent advance on HBOCs has favored to encapsulate Hb, aiming at mimicking the physiologically shielding effect of the erythrocyte membrane. Hb encapsulation within micro‐ or nano‐sized platforms offers great advantages. First, it mimics the encapsulation of Hb within native erythrocytes, which is closer to physiological environment.^[^ ^12^ ^]^ Second, the direct contact of Hb with plasma is avoided to prolong circulation, reduce uptake by the MPS and hypertensive response.^[^ ^61^ ^]^ Last, encapsulation strategy allows HBOCs to integrate other functional molecules such as allosteric effector molecules or antioxidant enzymes for better biological performance.^[^ ^64^ ^]^ Generally, Hb's encapsulation within various carriers such as liposomes and polymer particles could construct more suitable conditions for maintaining their functionality while preventing from extravasation and dissociation.^[^ ^61^ ^]^ Encapsulation of Hb with liposomes, cell membrane‐derived vesicles, and polymersomes, namely Hb vesicles (HbV), resembled cellular structure of erythrocytes.^[^ ^65^ ^]^ HbV performs similar O_2_ affinity to native erythrocytes (p50 = 28 mmHg) and shows no colloid osmotic pressure when suspended in saline solution, similar to erythrocytes.^[^ ^66^ ^]^ Another benefit of HbV is that their size (230–280 nm) is smaller than that of erythrocytes (≈8 µm), indicating better tissue permeability. The promise of HbV lies in their long half‐life, negligible infectious risks, and ease of accessibility.^[^ ^65^ ^]^ Another effective solution is loading Hb onto particles.^[^ ^12^ ^]^ Hb is loaded into the NPs or microparticles (MPs) by electrostatic adsorption, physical wrap, chemical coupling, and so on.^[^ ^12^ ^]^ This type of HBOCs is easier to purify and possess adjustable size and wider size range. Due to better mechanical capability, they could perform as microscaffold and provide area for cell adhesion, growth, and proliferation as well. These HBOCs have been mainly developed to provide extra oxygen for wound healing and tissue regeneration.

## 3 Erythrocyte Morphology Inspired Particles

Morphology, an essential property of particles, plays a vital role in the function of particles, including mitigating cellular responses and phagocytosis by macrophages in biomedicine.^[^ ^14^ ^]^ Considering this, the novel biomimetic particles pursue to resemble 3D structure of biological entities. Erythrocyte is one of the most popular imitated cell types because of their unique biconcave discoid morphology and mechanical flexibility.^[^ ^13^ ^]^ The hemodynamic distribution of erythrocytes in the blood flow is determined by their size, morphology, and flexibility. In middle and large blood vessels, erythrocytes flow along the central axis of vessels, while distributing throughout the vessels in the small capillaries for efficient O_2_ exchange.^[^ ^9^ ^]^ Besides, erythrocytes could avoid filtration in the spleen due to their biconcave discoid shape.^[^ ^8^ ^]^ The mechanical flexibility of erythrocytes allows them to deform into bowl shape to pass through the walls of blood vessels and the same deformation occurs when erythrocytes are cultured in proper hypertonic solution.^[^ ^10^ ^]^

Inspired by erythrocytes, non‐spherical particles mimicking the biconcave discoid features may show great potentials in biomedical applications. Explored particles mimic pivotal features, but not necessarily all details of native erythrocytes, to provide a greater diversity of biomaterials. Both experimental studies and theoretical investigations have already pointed to the benefits on vascular dynamics and cellular internalization.^[^ ^13^ ^]^ Non‐spherical particles might tumble or align in the flow process, which could reduce clearance by spleen or liver.^[^ ^67^ ^]^ According to their morphology, the erythrocyte‐like particles (ELPs) include discoid shaped, bowl shaped, ring shaped, and biconcave discoid shaped ones, resulting in different physical properties and biological performance. Among various ELPs, biconcave discoid particles are the most similar to erythrocytes morphology, and discoid MPs can be considered as a simplified form. Bowl‐shaped particles, similar to deformed erythrocytes, own unilateral axial concave structure on the basis of discoid MPs. The particles with ring shape can be regarded as the extreme case of radial complete depression of bowl‐shaped particles. The incorporation of biomaterial peculiarity with biological entities features would enable the ELPs to achieve unique and fascinating properties that spherical particles may be unable to possess.

Two main strategies have been developed to produce these NPs/MPs using various biomaterials. One is the template‐based strategy combined with replication or sacrifice of template, deformation, self‐assembly, and surface layer‐by‐layer (LbL) assembly. Another involves microfluidics or electrospray combined with self‐assembly of NPs induced by environments and the deformation of spherical particles. The former could precisely control the quality but is usually complicated, while the latter is simpler but less exactly controllable. Microfluidics and electrospray are advanced techniques for producing monodispersed droplets, which have been applied in the rapid and efficient preparation of non‐spherical particles. Most of them adopt one‐step, inexpensive, and versatile strategies, which allow them to prepare particles in various morphology through adjusting a battery of tunable experimental parameters. During the process, intervenention methods in the self‐assembly and deformation process mainly include solvent evaporation, diffusion, or extraction. The chemical reactions include electrostatic action, collapse caused by component dissolve, and so on.

### 3.1 Discoid Particles

Discoid particles inspired by erythrocytes have received increasing attention for their fascinating properties. In geometry, discoid micro/nano particles own high aspect ratio compared to spherical particles, which provides more target sites to contact and interact.^[^ ^68^ ^]^ In reality, the local geometry of particle decided whether macrophages initiate internalization instead of the overall particle shape.^[^ ^69^ ^]^ A seminal work reported that discoid particles might have a prolonged lifetime for the changed contact point between particles and cells‐inhibited macrophagic uptake.^[^ ^70^ ^]^ Major axis of these particles makes it less phagocytized by macrophages, causing a lower uptake in liver and prolonged navigation time in the blood.^[^ ^71^ ^]^ Besides, previous investigations also reported that the discoid particles exhibited a higher tendency to migrate to the wall during flow process.^[^ ^72^ ^]^ Despite discoid particles owning many advantages, only a few methods have been developed to fabricate discoid particles in a versatile and facile manner. The current methods are still somewhat confined by complexity of procedures, shape polydispersity, low yield, and high cost. The comprehensive summaries of reported fabrication strategies are introduced below.

**Figure 3:**
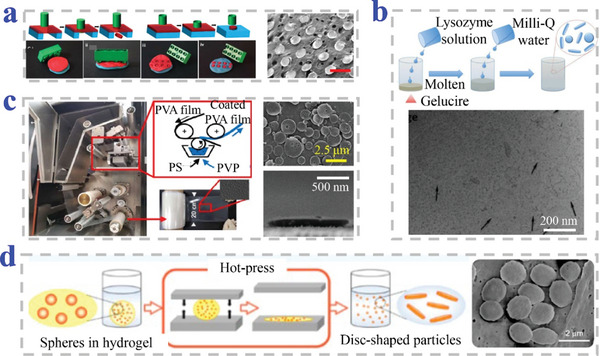
Preparation strategy of discoid particles based on template combined methods. a) Replication from template by MPP, where scale bar is 600 µm. Reproduced under the terms of the CC‐BY license.^[^ ^73^ ^]^ Copyright 2020, The Authors, Published by MDPI. b) Mechanical fragment of Gelucire 50/13 bulk gel in water. Reproduced with permission.^[^ ^74^ ^]^ Copyright 2015, Elsevier. c) Replication from roll‐to‐roll machine combined with phase separation. Reproduced with permission.^[^ ^75^ ^]^ Copyright 2019, Elsevier. d) Sacrificial matrix‐assisted hot‐press process. Reproduced with permission.^[^ ^76^ ^]^ Copyright 2020, American Chemical Society.

Keller et al. have used template‐based micromechanical punching (MMP) to fabricate flat, discoid poly(lactic‐co‐glycolic acid) (PLGA) MPs embedded with furosemide (**Figure** **3**a). The PLGA was prepared into a sheet carpeted on a deformable substrate and punched by a tough mold in MMP process to obtain the discoid morphology.^[^ ^73^ ^]^ Qi et al. proposed novel NPs prepared from fragmentation of stearoyl macrogol‐32 glycerides (Gelucire 50/13) hydrogels (Figure 3b). They have demonstrated the Gelucire 50/13 gels could be mechanically fragmented and deformed into stable NPs with discoid shape.^[^ ^74^ ^]^ Okamura et al. have developed two different methods to fabricate discoid particles. They employed roll‐to‐roll machine to fabricate polystyrene (PS) discs via phase separation of polymer in a continuous manner (Figure 3c). In this method, PS discs could be obtained in one pot by repeated procedures of washing and centrifugation collection.^[^ ^75^ ^]^ Then, they proposed hybrid discs of polymers by a sacrificial matrix technique assisted with hot‐press (Figure 3d). Hybrid MPs of PS, poly(L‐lactic acid) (PLLA), and poly(D,L‐lactide‐co‐glycolide) were embedded into alginate gel, creating space for deformation of MPs into individual discs in hot‐pressing process.^[^ ^76^ ^]^ Kharlampieva et al. designed discoid capsules fabricated from (methacrylic acid) (PMAA) multilayers on the sacrificial discoid silicon templates. The PMAA was sequentially assembled onto sacrificial substrates by LbL technique followed by pH‐induced dissolution of silicon templates.^[^ ^69^ ^]^

**Figure 4:**
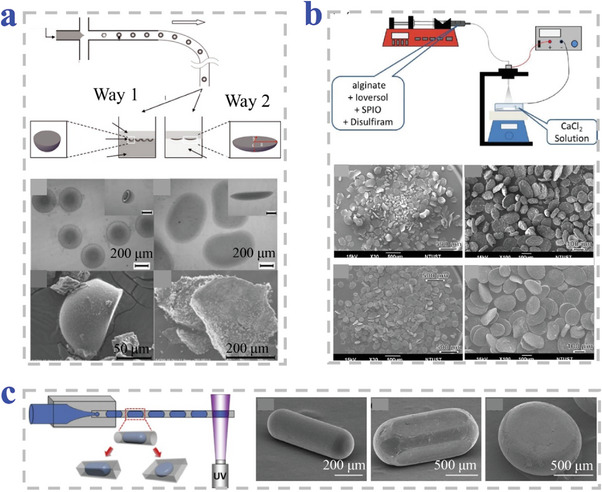
Strategies of microfluidic and electrospray combined methods. a) Microfluidic system‐assisted crosslinking at an oil/water interface. Reproduced with permission.^[^ ^77^ ^]^ Copyright 2012, Wiley‐VCH. b) Electrospray strategy assisted with agitation of gelling bath. Reproduced with permission.^[^ ^78^ ^]^ Copyright 2020, Wiley‐VCH. c) Microfluidic system of the spatial constraint of rectangular capillary. Reproduced with permission.^[^ ^79^ ^]^ Copyright 2014, Elsevier.

Lin et al. synthesized discoid chitosan MPs by microfluidic combined with deformation (**Figure** **4**a). Regular droplets produced in a cross‐junction channel were turned into discoid MPs by the following ionic gelation procedure at an oil/water interface in the collection pool.^[^ ^77^ ^]^ Bai et al. prepared disc‐like alginate MPs under the condition of agitation with gelling bath containing calcium chloride (Figure 4b). The oscillation of calcium chloride solution provided shear force to cause disc‐like morphology.^[^ ^78^ ^]^ In a previous research of our group, Cheng et al. fabricated discoid colloidal crystal MPs by microfluidic system (Figure 4c). Polyethylene glycol diacrylate (PEGDA) aqueous solution was used as dispersed phase of charged monodisperse silica (SiO_2_) NPs. Based on two‐phase coaxial microfluidic system, the photo‐polymerizing droplets templates were photo‐polymerized in a confined rectangular collection capillary. It was worth noting that the MPs with different sizes, color, and morphologies (discs, cuboids, and rods) could be produced by using different collection capillary in corresponding size and shape.^[^ ^79^ ^]^

### 3.2 Bowl‐Shaped Particles

The morphology of bowl‐shaped particles has obvious directional orientation for the concave structure with unidirectional sag. According to the radial depression degree, this kind of particles may include the concave shape with little depression and the parachute shape with large depression. The radial depression creates a unique cavity on particle, enhancing the deformation ability and providing a specific space to load drugs, fuel, and even cells. In addition, the sag side of MPs can stick tightly to the subject surface after the air in the cavity has been squeezed out by external forces, similar to suction cups. Although promising, there are small amount of developed bowl‐shaped particles and the preparation methods are generally limited.

**Figure 5:**
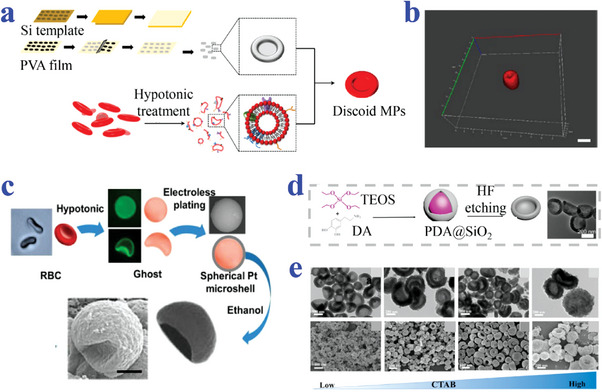
Strategies of template‐based methods. a,b) Replica molding from a sacrificial PVA template. Scale bar is 3 µm in (b). Reproduced with permission.^[^ ^80^ ^]^ Copyright 2018, Elsevier. c) Replication from erythrocyte ghosts as soft templates, scale bar is 2 µm. Reproduced with permission.^[^ ^10^ ^]^ Copyright 2019, Wiley‐VCH. d) Deformation of the PDA layers on sacrificial spherical template. Reproduced with permission.^[^ ^82^ ^]^ Copyright 2019, The Royal Society of Chemistry. e) Strategy of emulsion‐induced interfacial self‐assembly. Reproduced with permission.^[^ ^83^ ^]^ Copyright 2021, Elsevier.

Aryal and Key et al. manufactured silicon master template with an array of concave holes. Then, an intermediate polydimethylsiloxane template and eventually a sacrificial polyvinyl alcohol (PVA) template were generated via replicating from the former template. The polymeric mixture of PLGA was polymerized under UV light in the wells of the PVA template. The concave discoid particles were collected from PVA debris by filtration and centrifugation (**Figure** **5**a,b). A layer of erythrocyte membrane was then modified on the surface of the obtained particles to further simulate the characteristics of erythrocytes.^[^ ^80^ ^]^ Similarly, Decuzzi et al. reproduced concave MPs of a mixture of PLGA and PEG by replicating from a sacrificial PVA template.^[^ ^81^ ^]^ Han et al. used erythrocytes membranes as soft templates to prepare single‐sided concave discoid MPs under stimulation of hypertonic osmotic pressure (Figure 5c). The membrane ghosts were electroless plated with platinum and transformed into a bowl shape after treating with ethanol solution.^[^ ^10^ ^]^ Yan et al. synthesized hollow polydopamine (PDA) NPs via deformation of the PDA layers on sacrificial spherical template (Figure 5d). The core–shell templates were the PDA‐coated silicon NPs. The transformation of the core–shell to bowl‐like shapes was induced mainly by template dissolution and the osmotic pressure difference caused by hydrofluoric acid etching between the inner and outer sides.^[^ ^82^ ^]^ Wang et al. fabricated novel NPs by adopting strategy of emulsion‐induced interfacial self‐assembly (Figure 5e). Those polymer NPs underwent the surfactant‐templated sol–gel formation followed by in situ coating of mesoporous SiO_2_ crust. The obtained NPs possessed tunable concave degree morphology and surface roughness, which could be optionally tailored with the changed ratio of reactants.^[^ ^83^ ^]^

**Figure 6:**
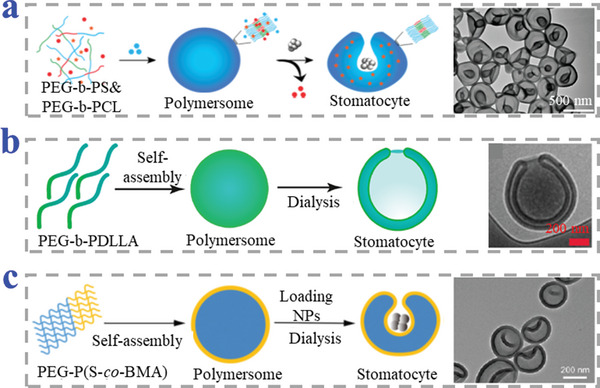
Amphiphilic block‐copolymers self‐assembly and transformation from spherical morphology to stomatocyte structure. a) PEG‐b‐PS and PEG‐b‐PCL. Reproduced with permission.^[^ ^98^ ^]^ Copyright 2017, American Chemical Society. b) PEG‐b‐PDLLA. Reproduced with permission.^[^ ^100^ ^]^ Copyright 2020, American Chemical Society. c) PEG‐P(S‐co‐BMA). Reproduced with permission.^[^ ^101^ ^]^ Copyright 2022, The Royal Society of Chemistry.

Amphiphilic block‐copolymers could assemble to polymersomes and the specific one containing a glassy hydrophobic segment could further induce a shape transformation from thermodynamically stable spherical morphology to kinetically trapped stomatocyte structure.^[^ ^84^, ^85^, ^86^ ^]^ In brief, the transformation from spherical shape to bowl‐shaped stomatocyte was driven by the osmotic pressure differences. Polymersomes of block‐copolymers with high glass transition temperature are flexible and responsive to external stimuli in the organic solvent. Dialysis of such structures induced osmotic pressure difference across the membrane, resulting in fast extrusion of plasticizing solvent and allowing distortion. When the solvent was completely removed, the hydrophobic segments in the polymersomes recovered their rigidity so that the stomatocyte structure was locked. So far, the used amphiphilic block copolymers include poly(ethylene glycol)‐block‐polystyrene (PEG‐b‐PS),^[^ ^84^, ^85^, ^87^, ^88^, ^89^, ^90^, ^91^, ^92^, ^93^, ^94^, ^95^, ^96^ ^]^ the disulfide bonds incorporated PEG block PS (PEG‐SS‐PS),^[^ ^97^ ^]^ mixture of poly(ethylene glycol)‐b‐poly(*ε*‐caprolactone) (PEG‐b‐PCL) and PEG‐b‐PS,^[^ ^98^ ^]^ poly (ethylene glycol)‐b‐poly(D,L‐lactide) (PEG‐PDLLA),^[^ ^99^, ^100^ ^]^ and poly(ethylene glycol)‐b‐poly(styrene‐co‐2‐hydroxy‐4(methacryloyloxy)benzophenone) (PEG‐P(S‐co‐BMA)),^[^ ^101^ ^]^ as shown in **Figure** **6**.

**Figure 7:**
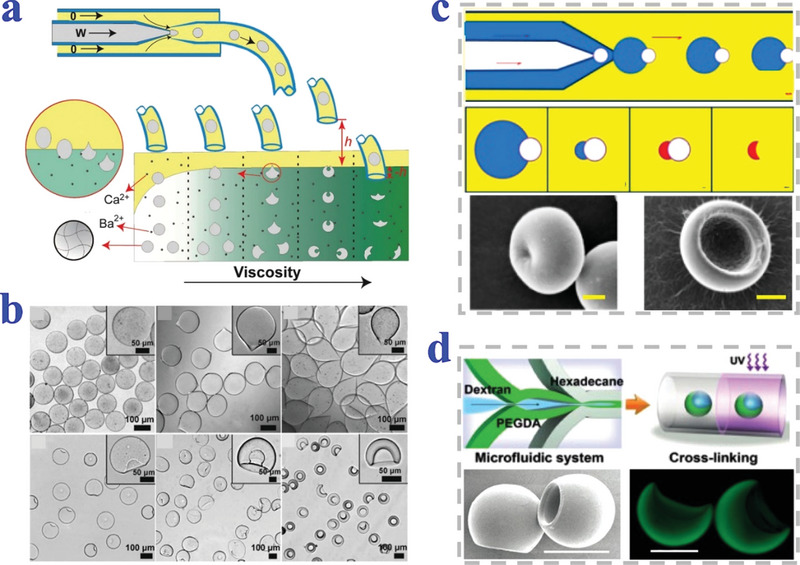
Strategy of microfluidic combined technique. a,b) Alginate microgels with varied shape by microfluidic technique. Reproduced with permission.^[^ ^102^ ^]^ Copyright 2012, American Institute of Physics. c) Bowl‐shaped MPs by attaching gas phase to the aqueous colloidal suspension droplets. Scale bars are 50 µm. Reproduced with permission.^[^ ^103^ ^]^ Copyright 2014, American Chemical Society. d) Bowl‐like MPs prepared by selective photo‐crosslinking of two‐phase component. Scale bars are 100 µm. Reproduced under the terms of the CC‐BY license.^[^ ^104^ ^]^ Copyright 2019, The Authors, Published by Wiley‐VCH.

Yang et al. have produced varied alginate microgels by microfluidic methods (**Figure** **7**a,b). The uniform emulsion droplets formed at the microcapillary were solidified by external ionic crosslinking to generate continuous fine tuning of MPs in the gelation bath under different gelation conditions. The gelation conditions involved viscosity of the gelation bath, collecting height and interfacial tension.^[^ ^102^ ^]^ Xu et al. attached a gas phase on the aqueous phase of colloidal suspension when generating the microdroplets in the double coaxial microfluidic system (Figure 7c). The attached microbubble on droplet created a tri‐phase interface and the shape of droplets could be adjusted by independently changing the flow rate of three phase solution.^[^ ^103^ ^]^ Similarly, Esch et al. fabricated bowl‐like MPs by selective photo‐crosslinking of one component in microfluidic device (Figure 7d). Specifically, dextran solution that cannot be photopolymerized was introduced into PEGDA solution that was capable of photopolymerization. The morphology was formed when PEGDA was selectively cross‐linked but dextran was removed. The size of cavity on MPs was controllable with altering the flow rate of each phase.^[^ ^104^ ^]^

**Figure 8:**
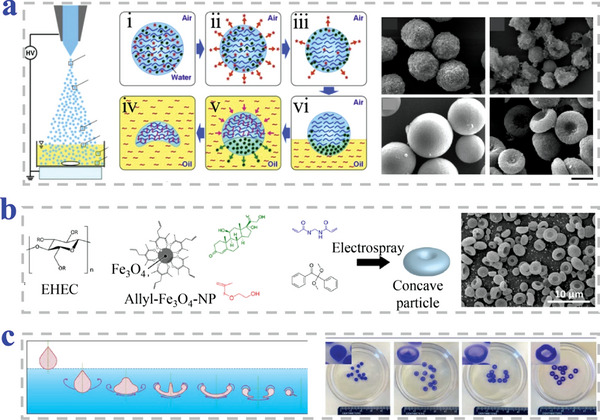
Strategies of electrospray combined method and vortex freeze. a) Electrospray combined solvent evaporation (scale bar: 5 µm). Reproduced with permission.^[^ ^105^ ^]^ Copyright 2016, Chinese Society of Particuology and Institute of Process Engineering, Chinese Academy of Sciences. Published by Elsevier. b) Electrospray technique combined intermolecular hydrogen bonds. Reproduced with permission.^[^ ^107^ ^]^ Copyright 2018, Elsevier. c) Electrospray‐assisted vortex ring. Reproduced under the terms of the CC‐BY license.^[^ ^108^ ^]^ Copyright 2016, The Authors, Published by Springer Nature.

Ju et al. successfully fabricated bowl‐shaped chitosan MPs by combining electrospray and solvent evaporation (**Figure** **8**a). The chitosan solution containing ethanol was used as the evaporable solvent while the diffusible solvent chosen was dimethyl sulfoxide (DMSO). Morphologies of the chitosan MPs were affected by the parameters of solvent diffusion process, including the volume ratio of evaporable solvent to diffusible solvent and the physical properties of the diffusible solvent. The chitosan MPs were endowed with acid‐responsive dissolution and performed auto‐fluorescence.^[^ ^105^ ^]^ Yogo et al. found that electrospray of the cellulose derivatives with *β*‐glucose yielded concave discoid particles (Figure 8b). The *β*‐glucose polymers might form concave discoid particles via the intermolecular hydrogen bonds of sheet structure. Ethyl(hydroxyethyl) cellulose (EHEC) with the *β*‐glucose structure was selected as precursor of the electrospray system and the particles experienced dented deformation in their center due to electrostatic repulsions of the surface charge and the undergone resistance from nozzle and collector.^[^ ^106^ ^]^ Furthermore, they have proved that the erythrocytes‐like MPs containing magnetite NPs could be synthesized using the electrospray technique as well.^[^ ^107^ ^]^

An et al. found the fluid would experience various intriguing geometrical intermediates such as spherical, concave discoid, and toroidal during a vortex ring process (Figure 8c). In a typical process, the vortex ring would occur when the droplet hit the free surface of a miscible solution at a sufficient impact speed. The droplets would experience deformation of curling back to dissipate the energy. Constantly changing intermediates of vortex ring‐derived particles (VRP) with varied shapes including the donut cap‐, jellyfish‐, and teardrop‐shaped particles were created. Intermediates were successfully captured by tuning the impact speed and viscosity of the droplets. They could be solidified into particles at controlled time points through gelation, crosslinking, or precipitation. More importantly, almost any materials, from polysaccharides (e.g., chitosan, alginate) to SiO_2_ colloids could be used once there existed a proper solidification event. Furthermore, electrospray technique could be utilized to produce the VRP with controllable shapes in large‐scale.^[^ ^108^ ^]^

Yang et al. reported a research that the presence of some additive (e.g., PVA, PEG) in the precursor solution, could effectively change the internal structures of the MPs in the electrospray process. The spherical polyethersulfone (PES) MPs prepared by the solution of PES and DMSO transferred into collapsed morphology when PEG was added to the PES and DMSO solution.^[^ ^109^ ^]^ Gao et al. successfully prepared erythrocyte‐like MPs by varying ratios of polylactic acid (PLA) and polyvinylpyrrolidone (PVP) during the electrospray process. When the ratios of PLA/PVP decreased, the spherical droplets of jet flow extended outward from the center to edge to form ELPs. The possible reason is that solvent evaporation accompanied by charge repulsion on the outside of the droplet was faster than the center while the microdroplets dropped from the nozzle to the collector. With rapid solidification of the droplets, a dent in the center of particle was acquired.^[^ ^110^ ^]^

**Figure 9:**
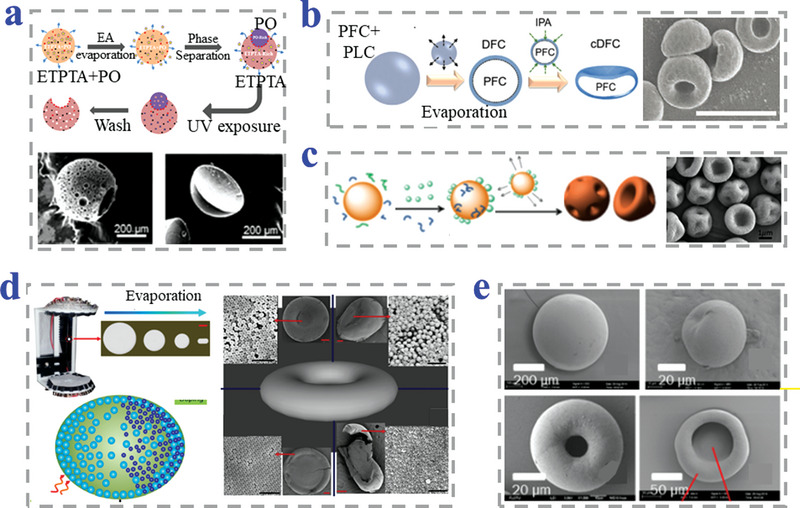
Strategies of phase separation combined methods. a) UV‐induced monomer polymerization in phase separation in microfluidic system. Reproduced with permission.^[^ ^111^ ^]^ Copyright 2019, American Chemical Society. b) The deformable core–shell particles fabricated by SPG membrane emulsification technique, scale bar is 10 µm. Reproduced with permission.^[^ ^112^ ^]^ Copyright 2021, Wiley‐VCH. c) Asymmetric shrinkage of a cross‐linked network during the phase separation process. Reproduced with permission.^[^ ^114^ ^]^ Copyright 2016, American Chemical Society. d) Self‐assembly of bowl‐shaped MPs combined with solvent evaporation. Reproduced with permission.^[^ ^115^ ^]^ Copyright 2019, Elsevier. e) Asymmetric shrinkage based on “coffee ring” effect during the phase separation. Reproduced under the terms of the CC‐BY license.^[^ ^116^ ^]^ Copyright 2016, The Authors, Published by MDPI.

Phase separation is an effective method to prepare anisotropic particles because of the separation between immiscible materials. Guo et al. fabricated monodisperse bowl‐like MPs in large quantities by phase separation (**Figure** **9**a). By changing the volume ratio of immiscible paraffin oil and ethoxylated trimethylolpropane triacrylate (ETPTA) in the initial emulsion, the resultant droplets template with the varied shape of nearly spherical, hemispherical to crescent‐shape could be obtained and then polymerized under UV light.^[^ ^111^ ^]^ Ito et al. developed novel deformable core–shell MPs of perfluorocarbon/poly(lactide‐co‐caprolactone) (PFC/PLC) by using the Shirasu porous glass (SPG) membrane emulsification technique (Figure 9b). The fabrication of concave‐shaped deformable particles involved phase separation induced by evaporation and subsequent solidification.^[^ ^112^ ^]^ Hosseinzadeh et al. synthesized erythrocytes‐like PS particles during the phase separation process. Ethylene glycol dimethacrylate and styrene were used as the cross‐linker and reaction medium, respectively. In the one‐pot dispersion polymerization process, the asymmetric shrinkage of polymerized network caused the bowl‐like morphology.^[^ ^113^ ^]^ Similarly, Zhang et al. proposed erythrocytes‐like PS particles in phase separation process due to the asymmetric shrinkage of polymerized network (Figure 9c). Divinylbenzene was utilized as cross‐linker while ethanol was chosen as reaction medium.^[^ ^114^ ^]^

In addition, the phase separation strategy has also been used to influence the self‐assembly process of NPs to prepare bowl‐shaped MPs. The main strategies in this method include solvent removal, solvent evaporation, and solvent extraction. Basu et al. designed bowl‐shaped MPs during the solvent evaporation process of suspended PS (Figure 9d). Evaporation flux varied along the droplet surface, leading to the equatorial diameter receding faster than the polar diameter, finally forming asymmetrical shape.^[^ ^115^ ^]^ Based on the influence of strong solvent extraction environment on the self‐assembly process of colloidal SiO_2_ NPs, namely “coffee ring” effect, anisotropic photonic crystal (PhC) MPs with varied shapes have been successfully prepared (Figure 9d).^[^ ^116^ ^]^ The fast extraction rate results in a lower diffusion rate of the colloidal NPs than the removal rate of solvent by extraction from the droplets. The resultant concentration of colloidal NPs was higher at the interface than that in the center during extraction. Subsequently, the polar parts of the droplets experienced continuous folding inward till the inner NPs completed assembly, finally forming the bowl‐like PhC particles. In the previous research of our group, Cai et al. has proposed bowl‐like PhC MPs by combining microfluidics and solvent extraction. In the process of rapid extraction of solvent and assembly of monodispersed NPs in droplets, PhC MPs of colloidal crystal clusters were formed. The research has demonstrated that the parameters of organic solubilities could influence not only the extraction rates to change formation time and the final morphology but also the order arrangement degree of the NPs.^[^ ^117^ ^]^ Furthermore, the PhC particles could be used as sacrifice template to produce hydrogel MPs. Photo‐crosslinked hydrogels could penetrate into the pores and be polymerized. Bowl‐shaped hydrogel MPs could be obtained after the SiO_2_ PhC templates were dissolved.^[^ ^118^, ^119^ ^]^

**Figure 10:**
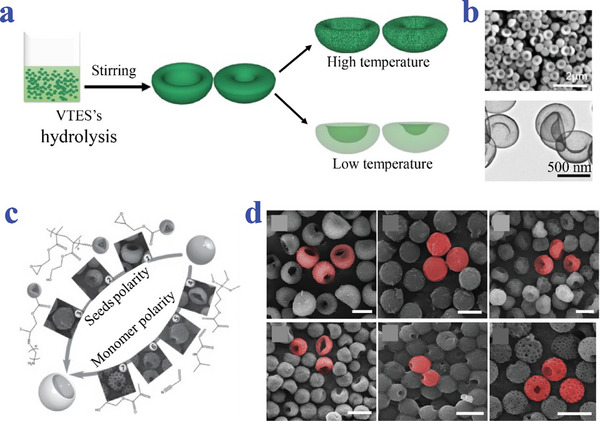
Special preparation methods for bowl‐shaped particles. a,b) The SiO_2_ NPs obtained by hydrothermal treatment. Reproduced with permission.^[^ ^120^ ^]^ Copyright 2016, Springer Science Business Media New York. c,d) The single‐hole PGMA swelling seed particles with microcavity controlled by the polarity of seed surface. Scale bars are 5 µm. Reproduced with permission.^[^ ^121^ ^]^ Copyright 2015, Wiley‐VCH.

There are still special methods for synthesizing bowl‐shaped particles. Wan et al. used potassium hydroxide to form the asymmetrical SiO_2_ NPs through quick condensation in rotation system (**Figure** **10**a,b). The uniformly hollow SiO_2_ NPs could be obtained by further hydrothermal treatment. This new approach relied on the consecutive hydrolysis and the condensation of vinyltriethoxysilane (VTES). Self‐hydrolysis of VTES was employed for the fabrication of erythrocyte‐like SiO_2_ NPs with high mono‐dispersity.^[^ ^120^ ^]^ Zhang et al. fabricated microcavities of polymer, which realized adjustable surface roughness and openings via single‐hole swelling seed particles of poly(glycidyl methacrylate) (PGMA) (Figure 10c,d). The morphology of PGMA/poly(styrene‐methacrylic acid) MPs from microcavity to erythrocyte shape was controlled by the polarity of seed surface.^[^ ^121^ ^]^

### 3.3 Ring‐Shaped Particles

Ring shape is also considered as torus shape or donut shape. Compared with the normal spherical particles, the ring‐shaped particles have several prominent superiorities, including but not limited to a higher ratio of surface to volume, better deformability, and a shorter path inside. The previous research has reported that artificial ring‐shaped particles were solely detected in the liver region without adverse effects and not found in other organs when they were intravenously injected into a mouse.^[^ ^122^ ^]^ These functional ring‐shaped particles are promising in many biomedical applications such as catalysis, cell encapsulation, therapeutic delivery, tissue engineering, and structural materials construction. Ring‐shaped particles have been produced in several previous works by adopting the strategies of spray drying, consolidation in microfluids, self‐assembly of colloid NPs on superhydrophobic surfaces or hydrophobic liquid, phase separation dispersion during polymerization, and so on.^[^ ^123^ ^]^ It is worth noting that coffee ring effect and vortex ring freezing technology can also produce ring‐shaped particles by changing the external conditions.^[^ ^108^, ^116^ ^]^

**Figure 11:**
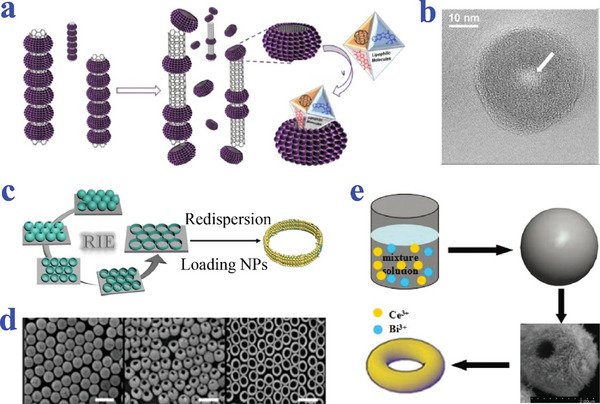
Preparation methods of ring‐shaped particles. a,b) Self‐organization and slip‐out on single‐walled carbon nanotube. Reproduced with permission.^[^ ^124^ ^]^ Copyright 2013, American Chemical Society. c,d) The SiO_2_ nanorings induced by deformation of hollow SiO_2_ particles by reactive ion etching. Reproduced with permission.^[^ ^125^ ^]^ Copyright 2016, American Chemical Society. e) The ring‐shaped particle of Bi_2_WO_6_@CeO_2_ synthesized through alcoholysis and subsequent facile calcinations. Reproduced with permission.^[^ ^126^ ^]^ Copyright 2014, The Royal Society of Chemistry.

Khiar et al. fabricated a new class of ring‐shaped NPs named glyconanosomes (GNSs) (**Figure** **11**a,b). GNSs were obtained by self‐organization and photopolymerization of glycolipid on single‐walled carbon nanotube and then slid out upon side wall.^[^ ^124^ ^]^ Clays et al. reported a new type of SiO_2_ nanorings induced through deformation of hollow SiO_2_ NPs in reactive ion etching process (Figure 11c,d). The resultant SiO_2_ nanorings could be utilized as universal templates to fabricate further ring‐shaped core–shell nanostructures when dispersed in solution.^[^ ^125^ ^]^ Guo et al. produced ring‐shaped particles of Bi_2_WO_6_@CeO_2_ through an environmental route and subsequent facile calcinations (Figure 11e). The alcoholysis of Bi^3+^ and Ce^3+^ results in Bi_2_WO_6_@CeO_2_ layers with nanometer precision. Meanwhile, carbonization of sucrose occurs and carbon‐Bi_2_WO_6_@CeO_2_ MPs will in situ form during solvothermal alcoholysis process. Subsequently, Bi_2_WO_6_@CeO_2_ composites are calcinations, leading to ring‐shaped hybrid NPs aggregates.^[^ ^126^ ^]^

**Figure 12:**
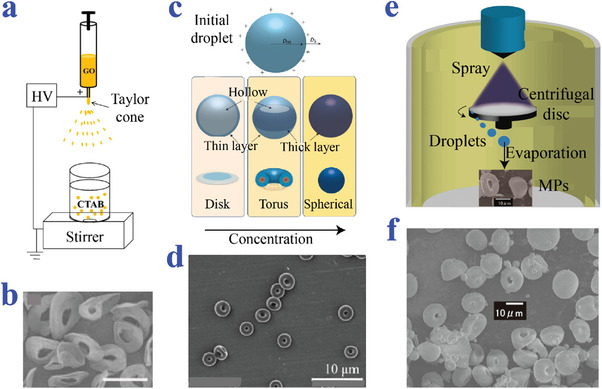
Preparation methods combined with electrospray. a,b) The ELGMs produced via electrospray‐assisted self‐assembly. Scale bar is 100 µm in (b). Reproduced under the terms of the CC‐BY license.^[^ ^127^ ^]^ Copyright 2013, The Authors, Published by Springer Nature. c,d) The particles prepared by controlling asymmetry diffusivities consolidations of charged droplets. Reproduced with permission.^[^ ^123^ ^]^ Copyright 2011, Elsevier. e,f) The donut‐like particles prepared by adopting a combined method of centrifugal disc atomization and spray drying. Reproduced with permission.^[^ ^128^ ^]^ Copyright 2018, The Chemical Society of Japan.

Chen et al. proposed novel erythrocyte‐like graphene microparticles (ELGMs) via electrospray assisted self‐assembly (**Figure** **12**a,b). Graphene oxide (GO) suspension was electrosprayed into cetyltrimethylammonium bromide (CTAB) coagulation bath, followed by chemical reduction to form ELGMs. Those erupted negative charged GO sheets droplets by electrospray were collected by CTAB solution with positively charged ammonium ions as coagulation bath. Electrostatic attractions between GO sheets and CTAB molecules took place to coagulate and stack from the bottom to the top of GO droplets. As a result, the top of GO coagulated shell was the thinnest and weakest, which caused the top layer collapse of particles. The droplets finally turned into ELGMs.^[^ ^127^ ^]^ Lee et al. successfully synthesized ring‐shaped particles through controlling asymmetry diffusivities and consolidation of charged droplets (Figure 12c,d). The torus structure was induced by fast solvent removal, which was controlled by the solvent type, surrounding temperature, flow rate, and concentration.^[^ ^123^ ^]^ Sakuma et al. fabricated uniform particles with donut‐like morphology adopting a combined method of centrifugal disc atomization and spray drying (Figure 12e,f). Clay fluid was driven by nitrogen gas and sprayed onto the rotating disc. The scattered droplets were dried on the disc to fabricate clay particles. Whether the tetrasodium pyrophosphate was added into the aqueous hectorite dispersion played a critical role in the change of particles in shape, size, and distribution.^[^ ^128^ ^]^

### 3.4 Biconcave Discoid Particles

**Figure 13:**
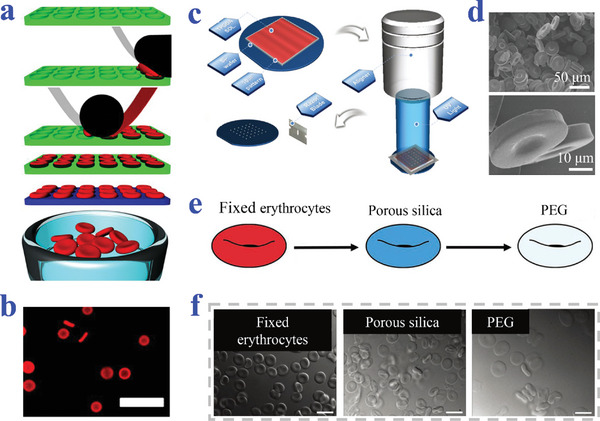
Preparation strategies of biconcave discoid particles based on template‐combined method. a,b) Replication from nonwetting templates of fluoropolymer molds. Scale bar is 20 µm in (b). Reproduced under the terms of the CC‐BY license.^[^ ^67^ ^]^ Copyright 2011, The Authors, Published by National Academy of Sciences. c,d) Method of moldless fabrication using a photopolymerization technique. Reproduced under the terms of the CC‐BY license.^[^ ^129^ ^]^ Copyright 2014, The Authors, Published by IOP Publishing. e,f) Shape‐preserved transformation from erythrocytes to hydrogel MPs. Scale bars are 10 µm in (f). Reproduced with permission.^[^ ^130^ ^]^ Copyright 2019, Wiley‐VCH.

The biconcave discoid particles are the most similar to the erythrocytes in morphology, which are considered to have high application prospects. They offer a paradigm for the construction of therapeutic and imaging agents delivery systems as they may combine versatility of synthetic biomedical particles and obtain broad functionality of natural erythrocytes. DeSimone et al. fabricated particles with similar characteristics in shape, size, and deformability to erythrocytes through replication from nonwetting templates of fluoropolymer molds (**Figure** **13**a,b).^[^ ^67^ ^]^ Song et al. proposed a moldless fabrication method combined with a photopolymerization technique to produce dimpled MPs (Figure 13c,d). The fabrication of MPs using monomer solution of tripropylene glycol diacrylate just needed standard UV lithography equipment and a film mask without mold.^[^ ^129^ ^]^ Kaehr et al. reported a strategy of shape‐retained transformation of biological erythrocytes into hydrogel MPs (Figure 13e,f). The external and internal surfaces of erythrocytes were chemically fixed and the resultant erythrocytes were replicated into conformal SiO_2_ layer in a sol–gel process. Shape‐retaining mesoporous SiO_2_ replicas were obtained when the template erythrocyte was removed. PEG precursors subsequently infiltrated into replicas and were cross‐linked before the SiO_2_ template was dissolved, obtaining final soft hydrogel replicas of erythrocytes.^[^ ^130^ ^]^

**Figure 14:**
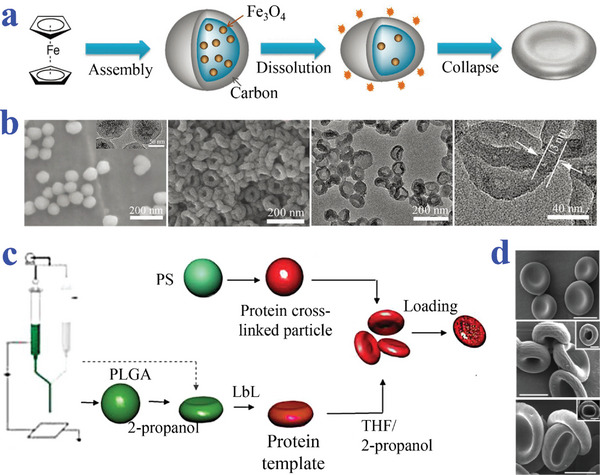
Preparation strategies of biconcave discoid particles based on collapse of spherical template. a,b) NPs obtained by dissolution of core of Fe_3_O_4_/carbon core/shell template. Reproduced with permission.^[^ ^131^ ^]^ Copyright 2019, Wiley‐VCH. c,d) ELPs from templates of PS or PLGA MPs. Scale bars in (d) are 50 µm (insets: 2 um). Reproduced under the terms of the CC‐BY license.^[^ ^132^ ^]^ Copyright 2009, The Authors, Published by National Academy of Sciences.

Zhang et al. synthesized biconcave carbon NPs by dissolving the magnetic core of a core/shell Fe_3_O_4_/carbon NP template (**Figure** **14**a,b). After the dissolution of Fe_3_O_4_ in an acidic and ethanol mixed solution at 200°C, the carbon shell was forced to collapse into a biconcave shape by the high pressure of ethanol gasification.^[^ ^131^ ^]^ Mitragotri et al. formed biconcave particles via collapse of hollow PS MPs upon solvent‐ or heat‐induced fluidization (Figure 14c,d). As an alternative, spherical PLGA particles could be prepared and the formation of erythrocyte‐like shape was induced when immersed in 2‐propanol. Particle surface was further modified through LbL technology, enabling the sequential electrostatic self‐assembly of cationic and anionic polymers. After the deposition of multiple layers, glutaraldehyde was used to crosslink the polymers shell to enhance stability of particles. The resultant particles were then incubated with tetrahydrofuran (THF) to remove template core and induce further collapse in center.^[^ ^132^ ^]^

She et al. fabricated biconcave discoid polyelectrolyte microcapsules through sacrificial template of Ca(OH)_2_. Template of Ca(OH)_2_ was obtained by rapidly pouring NaOH solution into CaCl_2_ solution dissolved with dextran sulfate (DS) and experiencing ultrasound. The precipitated Ca(OH)_2_ particles were biconcave discoid MPs to serve as templates. The assembly of polyallylamine and glutaralde occurred on the surface and then the templates were dissolved in a HCl solution, yielding hollow capsules.^[^ ^133^ ^]^ Similarly, Gu et al. synthesized biconcave discoid MPs of Hb–Ca(OH)_2_ that resembled erythrocytes in size and shape by co‐precipitation of Ca(OH)_2_ and Hb. The inorganic Ca(OH)_2_ MPs were fabricated almost as above but dissolving Hb into CaCl_2_ solution. During co‐precipitation process, Hb molecules were captured on the particle surface due to high adsorption capacity of DS.^[^ ^134^ ^]^ Huang et al. synthesized erythrocyte‐like Cu_1.8_S NPs via an ecofriendly one‐step solvothermal reaction with PVP.^[^ ^135^ ^]^ Zhang et al. reported a facile and controllable protocol to synthesize hierarchical erythrocyte‐like NPs of mesoporous SiO_2_, which were endowed with multi‐stack structure by using 11‐mercaptoundecanoic acid (MUA) and CTAB as co‐surfactants. These unique hierarchical structures of particles could be easily tuned when the molar ratios of CTAB to MUA were changed.^[^ ^136^ ^]^

**Figure 15:**
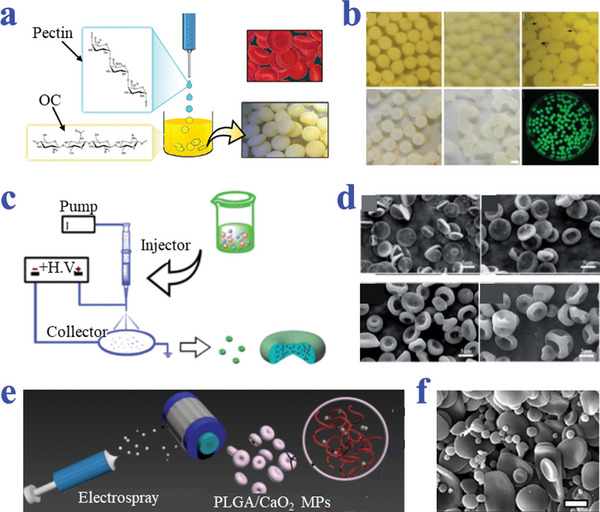
Preparation strategies of biconcave discoid particles based on electrospray‐assisted methods. a,b) The OC served as a non‐ionic cross‐linker of pectin to form erythrocytes‐shaped hydrogel capsules. Scale bars are 2 mm in (b). Reproduced with permission.^[^ ^137^ ^]^ Copyright 2014, Wiley‐VCH. c,d) The composite particles of Fe_3_O_4_/TbLa_3_(Bim)_12_/ PLGA prepared by electrospray technique. Reproduced with permission.^[^ ^139^ ^]^ Copyright 2018, The Royal Society of Chemistry. e,f) The PLGA/CaO_2_ MPs prepared via an electrospray process. Scale bar is 5 µm. Reproduced with permission.^[^ ^140^ ^]^ Copyright 2019, Wiley.

Zhang et al. discovered that oligochitosan (OC) could function as a non‐ionic cross‐linker to gel the pectin to form erythrocytes‐shaped hydrogel capsules (**Figure** **15**a,b). Chitosan and pectin have complementary charges and could form polyelectrolytes at the proper pH. In their study, the droplets of pectin solution were formed by extrusion and successfully crosslinked by ionic interaction when dropped into the OC solution. The morphology of hydrogel MPs strongly relied on concentrations of both OC and pectin.^[^ ^137^ ^]^ They further explored whether the stability of the particles could be improved and used to load Hb.^[^ ^138^ ^]^ Fan et al. prepared erythrocytes‐like composite particles of Fe_3_O_4_/TbLa_3_(Bim)_12_/PLGA by using electrospray (Figure 15c,d). Biconcave discoid particles could be successfully obtained when the precursor solutions with PLGA, Fe_3_O_4_, and TbLa_3_(Bim) were electrosprayed with THF as solvent. The difference of composite particles in shape was most likely attributed to the changed surface tension of the precursor solution. The jet flow with high surface tension more tended to form spherical particles while the one with the low surface tension collapsed to form erythrocytes particles.^[^ ^139^ ^]^ Karkhaneh et al. proposed novel PLGA/CaO_2_ MPs as oxygen carriers via an electrospray process (Figure 15e,f). Synthesized calcium peroxide (CPO) NPs were suspended in PLGA solution and PLGA was dissolved in dichloromethane. The solution was treated with electrospray to aluminum foil covered static collector and the derived particles were prepared after complete solvent removal.^[^ ^140^ ^]^

## 4 Biomedical Applications of Erythrocyte‐Inspired Materials

### 4.1 Drug Carriers for Therapies

#### 4.1.1 Cellular Therapeutics

Erythrocytes are considered as the most abundant cargo carriers in the blood, which make them inherently suited for intravascular delivery in vivo.^[^ ^141^ ^]^ Functionalized erythrocytes, the whole‐cell therapeutic delivery carriers, have been optimized as an advanced alternative to the traditional drug delivery system carriers because of their intrinsic biocompatibility, bioavailability, prolonged circulation, and remarkable stability in vivo.^[^ ^22^ ^]^ A mass of enzymes, proteins, micromolecular drugs, macromolecules, NPs, and even bacteria has been loaded to the surface or interior of erythrocytes, as mentioned in Section 2.1.^[^ ^8^, ^142^, ^143^, ^144^ ^]^ Their applications involve chemo/phototherapy for cancer,^[^ ^15^ ^]^ therapeutic delivery for antidotes,^[^ ^145^ ^]^ and binding of bacteria to membranes for anti‐infection.^[^ ^142^, ^146^ ^]^ They aim to modulate immune responses and to improve and alter pharmacokinetics of therapeutic.^[^ ^8^ ^]^ To be specific, the desirable erythrocyte‐based systems are characterized as follows: i) They can prevent drugs from degradation or inactivation, performing longer blood circulation. ii) The accessible regions are intra‐vascular or extravascular compartments accessible to erythrocytes. iii) The drug clearance pathway of therapeutic carried by erythrocytes is shifted from renal filtration in kidney to bile excretion in liver, typical degradation mechanism of Hb. iv) They can pass through the physiological barrier of human body and regulate immune response of immune system to cargoes. However, the utility of the whole‐erythrocyte as carrier system has also been seriously hindered due to some drawbacks including contamination problems and blood‐match needs.^[^ ^147^ ^]^ Besides, several disadvantages of technology remained in strict in vitro storage conditions, complex manufacture, and loading procedures, while the higher concentration and longer exposure time of partial types of NPs on erythrocytes would induce hemolysis and morphological changes of erythrocyte.^[^ ^148^ ^]^ Thus, ongoing advances are desired to meet the demand of clinical programs.

Instead of healthy erythrocytes, erythrocyte‐based gel has been reported as a great drug delivery system as well. Wang et al. reported that homologous erythrocytes could form the hydrogel when they were subcutaneously injected.^[^ ^149^ ^]^ In this system, blood clot was gel‐like fibrin network trapping mass of erythrocytes, which was formed by coagulation cascade process of ex vivo blood.^[^ ^150^ ^]^ Based on deep reddish color, the fabricated gels presented photothermal conversion under irradiation of NIR laser.^[^ ^151^ ^]^ Except for the role of drug delivery, they could recruit lots of immune cells, including B cells, macrophage, and T cells.^[^ ^150^ ^]^ Upon implantation, erythrocyte‐based gels have showed curative effective to treat rheumatoid arthritis,^[^ ^149^ ^]^ enhance bone repair,^[^ ^151^ ^]^ and for cancer immunotherapy and cancer vaccination.^[^ ^150^, ^152^ ^]^

#### 4.1.2 Membrane‐Derived Carriers

Going one step further, the arisen research mainly focuses on erythrocyte‐derivative systems, especially EM‐NVs. EM‐NVs have been implemented as “stealth” coating to camouflage drugs or synthetic NPs because they inherit specific biological functions of the source erythrocytes to evade immune elimination,^[^ ^30^ ^]^ and prolonged circulation time of therapeutic.^[^ ^1^, ^153^ ^]^ The EM‐NVs have been utilized to directly encapsulate chemotherapy drugs, enzymes, proteins, polysaccharides, toxins, and genetic material.^[^ ^154^, ^155^, ^156^, ^157^ ^]^ The camouflaged synthetic NPs included polymer NPs (e.g., PLGA, liposomes),^[^ ^156^, ^158^ ^]^ metal NPs (e.g., gold, Fe_3_O_4_, hollow copper sulfide),^[^ ^41^, ^159^ ^]^ inorganic non‐metal NPs (e.g., mesoporous SiO_2_),^[^ ^160^ ^]^ albumin NPs (e.g., BSA), and nanogels.^[^ ^30^, ^161^ ^]^ It is noteworthy that there are little target ligands on the EM‐NVs to provide the navigation for cargoes to locate at the disease region.^[^ ^15^, ^31^ ^]^ The capacity of erythrocyte membranes to fuse with other membranes provides feasible solutions through forming hybrid vesicles. The hybrid vesicles are endowed with homing tendency derived from membrane protein to target the disease region.^[^ ^30^, ^41^ ^]^ The hybrid vesicles could not only inherit the advanced performance of both source membranes such as immune evasion and half‐life extension but also achieve targeting disease sites and adherence to target cells. For instance, in a work by Shen et al., after intravenous injection of hybrid vesicles, retention in the blood was still more than 11.5% and 8.9% at 24 and 48 h, while the one without erythrocyte membrane was less than 3.1% and 1.7%, respectively. Six hours after intravenous injection, the concentration of hybrid vesicles was also significantly higher than the control in tumor tissues.^[^ ^162^ ^]^ In the investigation of Liu et al., the hybrid vesicles of erythrocyte and tumor cells succeeded in retaining tumor antigens and presenting to antigen‐presenting cells.^[^ ^163^ ^]^ The hybrid vesicles of erythrocyte and leukocytes or platelets could be exploited to cross the blood–brain barrier (BBB) and penetrate toward the brain parenchyma to enhance their effect in central nervous disease treatment.^[^ ^164^ ^]^ In contrast, the seperate erythrocyte did not have a mechanism to naturally cross the BBB.^[^ ^161^ ^]^ Furthermore, the camouflage of hybrid vesicles could improve biodistribution of NPs, reduce the adhesion of unrelated natural cells (e.g., white blood cells), and selectively improve the accumulation of NPs in specific organs such as the spleen and liver.^[^ ^7^, ^31^ ^]^ Due to their advantageous superiorities, the integration of other functional cell membranes into EM‐NVs has shown great potential in biomedical applications.

It is well known that erythrocytes work mainly in the blood; so that EM‐NVs are considered as promising carriers to promote therapeutic effectiveness of diseases with a high influx of blood flow and direct occurrence in the bloodstream. Therefore, they are shining in therapies toward heart‐ and vasculature‐related diseases and tumors. Due to the abnormal formation and leakage of new vessels as well as the exerted metabolic demands of tumors, NPs could be preferentially located at the tumor sites for enhanced retention with more blood flowing through.^[^ ^20^, ^165^, ^166^ ^]^ EM‐NVs‐NPs have emerged as a promising administration under preclinical investigation to improve the efficiency of chemotherapy,^[^ ^167^ ^]^ photothermal therapy (PTT),^[^ ^168^ ^]^ sonodynamic therapy,^[^ ^169^ ^]^ radiation therapy, and immunotherapy.^[^ ^15^, ^170^, ^171^ ^]^ The therapeutic improvements of EM‐NVs‐NPs systems are mainly attributed to their enhanced blood circulation, improved immune evasion, and extended half‐life, which decrease toxicity of NPs and potentiate the uptake of tumor cells.^[^ ^161^ ^]^ Smaller size of EM‐NVs‐NPs also affords them the ability to penetrate deep into tissues and makes it more advantageous in cancer treatment than using erythrocyte as carriers.^[^ ^172^ ^]^ Surface functionalization of EM‐NVs by using targeting moieties could be utilized to enhance penetration of active drugs into tissue sites of solid tumor; thus, achieving potentiated curative effects.^[^ ^161^ ^]^ EM‐NVs have also focused on treatments of vasculature‐ and heart‐related disorders and autoimmune diseases,^[^ ^157^ ^]^ such as ulcerative colitis, inflammatory bowel disease, type 1 diabetes, and encephalomyelitis.^[^ ^161^ ^]^

Besides, the EM‐NVs have been specifically developed to treat the infectious diseases and organophosphate poisoning. Based on molecular affinity between erythrocyte membrane and pathogens/organophosphate, EM‐NVs could realize detoxification by adsorbing pathogens/organophosphate.^[^ ^161^ ^]^ Bacteria could secrete pore forming toxins (PFTs) to disrupt the membranes of specific host cells; thus, causing considerable damage to the host cells.^[^ ^49^ ^]^ EM‐NVs‐NPs are used as decoy to target toxins; and subsequently, to trap, neutralize, and clear target toxins from the body.^[^ ^173^ ^]^ Hu et al. have shown that EM‐NVs‐PLGA NPs prevented rats from death when premixed with Hl*α* (a kind of PFTs) prior to lethal intravenous injection.^[^ ^174^ ^]^ In another case of skin lesion induced by MRSA, the EM‐NVs could be localized to the regions where bacteria were colonizing and secreting the most toxins, synergistically detoxifying to enhance antivirulence efficacy.^[^ ^175^ ^]^ Besides, EM‐NVs could decrease antibiotic resistance through immune escape to improve their effectiveness at the infection microenvironment.^[^ ^176^ ^]^ Organophosphate exposure, another toxic action, is generally caused by the use of pesticide and insecticide like dichlorvos.^[^ ^49^ ^]^ Organophosphate could irreversibly phosphorylate acetylcholinesterase, causing organophosphate poisoning. Acetylcholinesterases on erythrocyte membrane could relieve toxicity by presenting decoy acetylcholinesterase, providing a novel way to target organophosphate to avoid organophosphate poisoning. In addition, the EM‐NVs could circulate in the blood in extended time to bind organophosphates and be safely metabolized in the liver.^[^ ^177^ ^]^

#### 4.1.3 Particle Carriers

Biomimetic particles are the bio‐inspired abiotic particles of morphology‐inspired particles or particles that mimic biological functions, which establish a bridge to combine the great advantages of erythrocyte property of natural selection and the materials performance.^[^ ^178^, ^179^, ^180^ ^]^ Through rational design, they could cause to reappear, biomimetic features and mechanism of a biomodulation process in a biological event.^[^ ^181^, ^182^ ^]^ These biomimetic particles give critical revelations for developing as therapeutics and better interfacing with biological systems, including modulating biological functions of cells, encouraging tissue regeneration, and enhancing traditional therapeutics delivery.^[^ ^183^ ^]^ ELPs have gained great interests due to their drug delivery potential, nonspecific clearance by RES,^[^ ^184^ ^]^ and enhanced targeted binding to cells.^[^ ^185^ ^]^ For better performance, some ELPs have also adopted hydrogels as substrates for their adequate flexibility and similar compressibility to erythrocyte.^[^ ^186^ ^]^ ELPs are considered as advanced drug carriers, attributed to the fact that the biconcave discoid shape provides a larger surface area than spherical particles. That is favorable for therapeutic encapsulation and release, while the shortened distance from the inner to the exterior of ELPs makes therapeutic release via diffusion more complete.^[^ ^13^ ^]^ Besides, ELPs with a larger radius of curvature increase the available functionalized area for protein/ligand modification and cell adhesion, increasing the binding affinity and cellular interaction compared with volumetrically equivalent spherical particles.^[^ ^187^, ^188^ ^]^

Another advantageous capacity of ELPs as drug delivery systems is that the release profile could be changed through the selection of different substrate biomaterial. Normally, drugs encapsulated within the particles are released with the particles degradation or diffusion, mainly depending on strategies of drugs encapsulation and biomaterials property thereof.^[^ ^189^ ^]^ ELPs composed of biomaterial with responsive or targeted degradation ability have been used as drug carriers for intelligent therapy. For example, ELPs of PMAA in response to pH stimuli have evolved over time and the erythrocytes‐like shape performed positive interaction with several cell types.^[^ ^69^ ^]^ Based on the previous introduction on the preparation of ELPs in Section 3, the following **Table** **1** has put the attention on synthetic ELPs that have been tested in practically applied research.

**Table 1:** Biomedical applications of ELPs with different morphologies for the purpose of therapy

Shape	Biomaterial	Size	Method	Drugs	Applications
Discoid	Alginate	MPs	Electrospray	Disulfiram–SPIO	Combined therapy on ovarian cancer^[^ ^68^, ^78^ ^]^
	PLGA	MPs/NPs	Replication from roll‐to‐roll machine	—	Enhanced adhesiveness for drug delivery^[^ ^75^, ^190^ ^]^
	PS, PLLA, PLGA	MPs	Sacrificial matrix‐assisted hot‐press	—	Enhanced adhesiveness for drug delivery^[^ ^76^ ^]^
	PLGA	MPs	Replication by MPP	Furosemide	Drug delivery applications^[^ ^73^ ^]^
	Gelucire 50/13	NPs	Mechanical fragment of gel in water	Protein	Macromolecule delivery^[^ ^74^ ^]^
	PMAA	MPs	Sacrifice of templates	—	Carriers with pH‐triggered transport in cancer therapy^[^ ^69^ ^]^
	Chitosan	MPs	Microfluidic emulsification	Ampicillin	Controlled drug release for pharmaceutical^[^ ^77^ ^]^
Bowl‐shaped	PLC	MPs	SPG membrane emulsification	PFC	Oxygen carriers for blood substitutes and tissue engineering^[^ ^112^ ^]^
	PLGA, erythrocyte membrane	MPs	Replica molding from a sacrificial PVA template	—	Drug delivery in blood circulation^[^ ^80^ ^]^
	GelMA	MPs	Microfludic‐assisted solvent extraction	Dexamethasone	Adhesive drug carrier for ulcerative colitis^[^ ^118^ ^]^
	GelMA, magnesium	MPs	Microfludic‐assisted solvent extraction	Ranitidine	Micromotors for ulcer of stomach^[^ ^119^ ^]^
	Mesoporous carbon, SiO_2_	NPs	Emulsion‐induced interfacial self‐assembly	5‐Fluorouracil	Nanomedicine carrier with pH‐responsive drug release and remarkable intracellular uptake^[^ ^83^ ^]^
	ETPTA, MnO_2_	MPs	Microfludic‐assisted phase separation	Fe_3_O_4_	Micromotor for degradation of methylene blue^[^ ^111^ ^]^
	PEGDA	MPs	Microfludic	Cell	Cell carriers for therapy^[^ ^104^ ^]^
	PLGA, PEG	MPs	Replicating from a sacrificial template	Tissue plasminogen activator	Enhanced lysis of blood clots^[^ ^81^ ^]^
	Chitosan	MPs	Electrospray	—	Carrier with acid‐triggered dissolution^[^ ^105^ ^]^
	PEG‐b‐PLC, PEG‐b‐PS, platinum	NPs	Self‐assembly of amphiphilic block‐copolymers	DOX	Biodegradable nanomotor^[^ ^98^ ^]^
	PEG‐SS‐PS, platinum	NPs	Self‐assembly of amphiphilic block‐copolymers	DOX	Redox‐responsive nanomotor for cancer therapy^[^ ^97^ ^]^
	PEG‐b‐PS, catalase	NPs	Self‐assembly of amphiphilic block‐copolymers	—	Nanomotor to penetrate across the vasculature and enhance uptake by cancer cells^[^ ^92^ ^]^
	PEG‐PDLLA, MnO_2_	NPs	Self‐assembly of amphiphilic block‐copolymers	—	Nanomotor with enhanced penetration behavior to 3D cell spheroids of HeLa cells^[^ ^100^ ^]^
	PEG‐b‐PS, iron oxide	NPs	Self‐assembly of amphiphilic block‐copolymers	Zinc phthalocyanine	Nanomotor for photodynamic therapy^[^ ^95^ ^]^
Ring‐shaped	Diacetylenic‐based lycolipid	NPs	Self‐organization and slip‐out	Camptothecin	Breast cancer^[^ ^124^ ^]^
	Nanoclay hydrogel	MPs	Deformation of hollow particles by etching	DNA or cells	Bioencapsulation and cell‐free protein production^[^ ^108^ ^]^
Biconcave discoid	Carbon	NPs	Deformation of core/shell template	DOX	Carrier with pH‐responsive drug release and chemo‐photothermal tumor therapy^[^ ^131^ ^]^
	PLGA	MPs	Electrospray	Calcium peroxide	Oxygen carrier to promote proliferation and osteogenic differentiation of MSCs cultured on MPs^[^ ^140^ ^]^
	Pectin, OC	MPs	Electrospray	Hb	Oxygen carrier^[^ ^137^, ^191^, ^192^ ^]^
	Silk fibroin	MPs	Replicating from template	—	Drug carrier targeting lung^[^ ^193^ ^]^
	Dextran, Ca(OH)_2_	MPs	Co‐precipitation	Hb	Oxygen carry^[^ ^134^ ^]^
	Mesoporous SiO_2_	NPs	Intermolecular interactions	DOX	DOX delivery and adsorption of rhodamine B and methylene blue^[^ ^136^ ^]^
	PLGA	MPs	Deformation of hollow particles	Hb/dextran/heparin	Drugs delivery carrier in blood^[^ ^132^ ^]^

SPIO: superparamagnetic iron oxide; GelMA: methacrylated gelatin; MSCs: mesenchymal stem cells; DOX: doxorubicin; MnO2: manganese dioxide.

### 4.2 Biological Sensor and Diagnostics

Although a mass of current research of erythrocytes or erythrocyte derivatives‐integrated carriers has emerged to deliver therapeutic agents, there exist less investigations about their applications in biosensor construction and diagnostic/imaging agent delivery. The advanced biosensors and bioimaging are promising for in situ sensing and imaging of biomolecules in individual living cells, medical diagnostics, and cellular activities, aiming at enhancing the stability, and biodistribution of imaging agents and biosensors.^[^ ^22^, ^194^ ^]^ Plasmonic noble metal nanomaterials (e.g., Ag, Au, and Au@Ag core–shell particle) could be utilized in localized surface plasmon resonance (LSPR) to sense molecular activities.^[^ ^22^, ^195^ ^]^ Au‐NPs are widely applied as optical markers for PTT of cancer due to their intense optical absorption and subsequent nonradiative energy dissipation. However, the application of those novel biosensors in clinical medicine is limited for the short half‐life time and fast phagocytosis in blood, tissues, and organs.^[^ ^196^ ^]^ Through camouflaging NPs with erythrocytes or membrane vesicles, obtained engineered erythrocytes and EM‐NVs‐NPs achieved prolonged circulation and better bioavailability.^[^ ^37^ ^]^ For instance, various functional metal–organic framework (MOF) NPs were assembled to native erythrocytes to sense nitric oxide in blood and application of other multimodal imaging.^[^ ^197^ ^]^ The EM‐NVs‐Au‐NPs performed long‐term and provided sensitive detection of tumor for preferential accumulation at the tumor site.^[^ ^198^ ^]^

Based on specific identification of pathogens to erythrocytes, EM‐NVs provided novel attempts for the diagnosis of infectious diseases.^[^ ^199^ ^]^ EM‐NVs‐NPs have been developed as combination detection approach for targeting and isolation of pathogen‐associated proteins, which have been investigated in pathogen schistosoma mansoni and pathogen group A streptococcus.^[^ ^200^ ^]^ Importantly, this detection approach has the potential to improve the distinguishing of both known and unknown virulence pathogens and antigens on immune cells. EM‐NVs have effectively labeled erythrocyte‐induced B cells and imaged acute inflammation by white blood cell labeling in murine models.^[^ ^201^, ^202^ ^]^ Interestingly, Liu et al. proposed erythrocyte‐camouflaging bacteria for bioimaging of cancer. The hybrid system reduced inflammatory response and prolonged reservation of bacteria but did not change inherent bioactivities in the disease sites. The luminescence signals could maintain for multiple days due to the reproductive performance of bacteria inside the tumor tissues.^[^ ^203^ ^]^

To get sufficient quality and observe minute details in captured images, it's essential to employ contrast agents to enhance the resolution and sensitivity of imaging, especially for X‐ray imaging, magnetic resonance imaging (MRI), and ultrasound imaging.^[^ ^153^ ^]^ Paramagnetic and superparamagnetic NPs have been broadly utilized as contrast agents to enhance the sensitivity and specificity of MRI. For instance, gadolinium oxide NPs and gadolinium hybrid NPs have been used as MRI probes in the early stage (from about the 1980s) and the biocompatible superparamagnetic iron oxide NPs (IONPs) have emerged as a better alternative to reduce fast liver accumulation renal failure in the patient.^[^ ^204^, ^205^ ^]^ Their encapsulation in erythrocytes or EM‐NVs further exhibited high relaxation rates and improved the pharmacokinetics and bioavailability.^[^ ^206^, ^207^ ^]^ Microbubbles are effective contrast agents of ultrasound imaging, but their application potential is limited due to poor tissue permeation, low stability, and fast clearance. Lu et al. presented a promising EM‐NVs‐NPs‐composed nanosized microbubble in which dodecafluoropentane core was coated by EM‐NVs as a contrast agent, called “Sonocyte”. Sonocyte performed superior tumor penetration and accumulation to improve the sensitivity and specificity of tumors identification in ultrasound assessment.^[^ ^208^ ^]^

Indocyanine green (ICG) could not only be utilized in clinic for photodynamic therapy (PDT) and PTT but also served as a fluorescence probe for imaging under NIR laser.^[^ ^209^ ^]^ Several EM‐NVs carrying ICG have been studied for imaging of varied tumors due to enhanced permeability and retention effect in tumors.^[^ ^210^, ^211^ ^]^ Besides, it was proved that the system of EM‐NVs‐ICG was also suitable for imaging of ischemic stroke.^[^ ^212^ ^]^ Liu. et al. have synthesized a core–shell zinc gallogermanate nanostructures‐mesoporous SiO_2_ and further enveloped by EM‐NVs. The nanostructures performed long‐term NIR luminescence stability and excellent biocompatibility for bioimaging in vivo and monitoring tumors.^[^ ^213^ ^]^ Besides, the EM‐NVs‐NPs loaded with upconversion NPs were used for tumor imaging due to reduced uptake of RES and extended circulation in blood. The upconversion NPs core could convert near‐infrared radiation to visible light, which was visible on MRI.^[^ ^214^ ^]^

**Figure 16:**
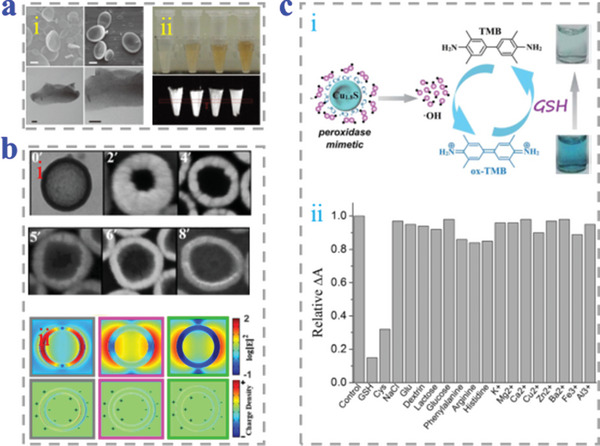
ELPs for detection and imaging. a) ELMs with performance in MRI. a‐i) SEM images and TEM images of ELMs. a‐ii) Performance of ELPs at different concentrations in MRI. Reproduced with permission.^[^ ^106^ ^]^ Copyright 2010, Wiley‐VCH. b) ELPs as a platform for biosensing. b‐i) Images of ELPs during the etching process. b‐ii) Comparison of a SiO_2_–Au core–shell particle and a solid Au nanoring for normal incidence illumination under the NIR. Reproduced with permission.^[^ ^125^ ^]^ Copyright 2016, American Chemical Society. c) ELPs as detection sensor of GSH. c‐i) Schematic illustration of the Cu_1.8_S NPs as a selective colorimetric detection sensor. c‐ii) The selective detection of the GSH in complex assay with several interference substances. Reproduced with permission.^[^ ^135^ ^]^ Copyright 2017, The Royal Society of Chemistry.

In addition to erythrocyte derivatives, the ELPs have also been studied for imaging purposes. Yogo et al. proposed ELMs encapsulated with fluorescent dyes and magnetite NPs, which exhibited effective fluorescence performance and dark contrast in MRI (**Figure** **16**a).^[^ ^106^ ^]^ Clays et al. designed ELPs which could load different metal NPs to serve as an interesting platform for biosensing. When Au NPs or IONPS were loaded, the ELPs showed high quality factor resonances in the NIR and magnetic hyperthermia in MRI, respectively (Figure 16b).^[^ ^125^ ^]^ Fan et al. fabricated composite ELPs of Fe_3_O_4_/TbLa_3_(Bim)_12_/PLGA for accurate drug tracking, which have shown excellent fluorescent properties and tunable magnetism.^[^ ^139^ ^]^ Huang et al. prepared nano‐sized ELPs of Cu_1.8_S with strong intrinsic peroxidase‐like activity as a selective colorimetric detection sensor for glutathione (GSH) (Figure 16c).^[^ ^135^ ^]^

### 4.3 Tissue Engineering

The erythrocyte‐inspired biomaterials used in tissue engineering primarily serve as oxygen carriers to provide extra oxygen, relying on HBOCs as mentioned in Section 2.3. The potential applications involve erythrocyte substitutes in blood transfusion and oxygen supply module in scaffold or cell culture in vitro. Blood transfusion meets the demands of acute trauma, anaemia, and surgical procedures but faces the inherent difficulties of erythrocytes such as short shelf life, infection, and blood type mismatching.^[^ ^215^ ^]^ The developed erythrocyte substitutes largely undertake the oxygen delivery role of erythrocytes for serving as oxygen delivery systems.^[^ ^216^ ^]^ HBOCs are promising erythrocyte substitutes because they integrate functional Hb of erythrocytes as oxygen delivery module. They are free of infectious agents, prolong shelf‐life, and are independent of the recipient blood type.^[^ ^217^ ^]^ The safe and affordable HBOCs have a promising potential on tissue perfusion and oxygenation for reconditioning of kidney grafts and pretransplant assessment. HbV with high biocompatibility, pathogen free, and high stability in vitro and in vivo are most anticipated in overcoming current obstacles of blood transfusion, especially the vesicles derived from erythrocyte membrane. For instance, Leticia et al. used MOF NPs loaded with Hb and camouflaged with EM‐NVs as potential oxygen delivery systems as a blood surrogate.^[^ ^215^ ^]^

HbV possess similar Hb concentration to native erythrocytes.^[^ ^218^, ^219^ ^]^ For metabolization, clearance of HbV was mainly by macrophages in the liver and spleen, similar to senescent erythrocytes.^[^ ^12^ ^]^ Besides, HbV could easily pass through collateral arterioles to peripheral tissues of the myocardial system due to their much smaller sizes compared to native erythrocytes.^[^ ^220^ ^]^ The serious drawbacks of HbV are the facts that they might cause peroxidation in injuries of ischemia reperfusion.^[^ ^221^, ^222^ ^]^ ELPs with oxygen delivery capacity provide novel great options as blood substitutes in hematology, blood doping, prolonging half‐time, and avoiding filtration in the spleen in the body. Gu et al. fabricated Hb‐based ELPs as oxygen carriers and electrochemically evaluated their ability of oxygen carrying.^[^ ^134^ ^]^ Zhang et al. developed ELPs of pectin‐based hydrogel and encapsulated Hb at the high efficiency of 99.99%.^[^ ^191^ ^]^ Ito et al. also fabricated concave shape oxygen carriers loaded with PFC instead of Hb to delivery oxygen, which could pass through a silicon microchannel with a 4.5 µm gap to enhance oxygen supply under hypoxic conditions.^[^ ^112^ ^]^ However, all researches of the above three groups have not further evaluated their performance in living body.

For in vitro 3D cell culture systems and in vivo scaffold in tissue engineering, one vital barrier is supplying adequate oxygen. The previous researches have reported that oxygen concentration serves as a significant signaling molecule to control differentiation of stem cell within tissue engineering scaffolds and affect both the final functionality and architecture of the restored tissue in vivo regeneration process.^[^ ^223^, ^224^ ^]^ For scaffold in vivo, the oxygen distribution is unequal for limited oxygen diffusion from surrounding vasculature to the periphery of scaffolds. The cells around the center of scaffolds that are nonvascularized have tendency to die due to lack of oxygen supply. Thus, the feasibility and effectiveness of current scaffolds are limited for regeneration of severe defected tissues.^[^ ^225^ ^]^ Therefore, the presence of a scaffold with local O_2_ supply module helps in accelerating the regeneration process by maintaining suitable oxygenation levels of tissue.^[^ ^224^ ^]^

**Figure 17:**
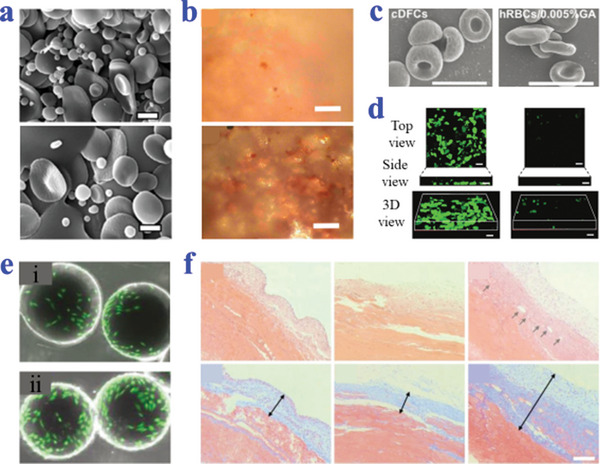
Erythrocyte‐inspired oxygen carriers and functional particles in tissue engineering. a,b) ELPs of PLGA/CaO_2_. SEM images of ELPs with or without CaO_2_ (a). Images of alizarin red stained cells cultured on ELPs with or without CaO_2_. Reproduced with permission.^[^ ^140^ ^]^ Copyright 2019, Wiley (b). c,d) ELPs of PFC as oxygen carriers. SEM images of formation process of ELPs (c). Images of the multilayered EGFP‐HeLa cells cultured at 2% O_2_ (left) and less than 2% O_2_, captured by confocal microscopy (d). Reproduced with permission.^[^ ^112^ ^]^ Copyright 2021, Wiley‐VCH. e,f) Oxygen carrier of GelMA MPs. The confocal laser scanning images of 3T3 cells cultured on different MPs with (down) or without Hb integrated (top) (e). The H&E staining (top) and Masson staining (down) of repaired tissues treated with MPs with oxygen, MPs, and without any treatment (from right to left) after implantation for 2 weeks (f). Reproduced with permission.^[^ ^231^ ^]^ Copyright 2019, Wiley‐VCH.

Various HBOCs have been utilized to supply O_2_ to maintain it in physiologically relevant levels, aiming at improving cell viability in tissue regeneration and 3D culture systems.^[^ ^226^ ^]^ However, the field is still in its infancy with few proved practical applications. The initial studies used Hb directly but the results indicated that the Hb oxidation affected cell behavior by upregulating genes associated with metabolism of reactive oxygen species.^[^ ^227^ ^]^ The subsequent attempts focused on minimizing contact of cell and HBOCs to reduce oxidative stress. Centis et al. employed fibrin gels to encapsulate cells; so that, the cells were separated from supplemented medium containing HBOCs. The results demonstrated that the separation could increase cell viability and downregulate gene expression of HIF‐1a in response to hypoxia.^[^ ^228^ ^]^ In this field, micron‐sized particles have promising applications for no need of intravenous administration and providing contact area for cell growth.^[^ ^226^, ^229^, ^230^ ^]^ Karkhaneh et al. fabricated amine‐decorated ELPs of PLGA/CaO_2_ with controlled oxygen release capacity.^[^ ^140^ ^]^ After integration of CaO_2_, the particles could persistently release oxygen and achieve content of 35–67.5 mmHg for bone tissue engineering. Compared to blank counterparts of PLGA, the MPs with CaO_2_ induced the proliferation of mesenchymal cells after cell seeding (**Figure** **17**a,b). Ito et al. developed PFC‐based oxygen carriers of ELPs for blood substitution and tissue engineering. The ELPs successfully passed through silicon microchannel at a 4.5 µm gap due to their diameter, shape, and low Young's modulus (93 KPa). They performed promoted oxygen supply to promote proliferation of cells in multiple layer under hypoxic conditions (Figure 17c,d).^[^ ^112^ ^]^ In the previous research of our group, Liu et al. used GelMA MPs integrated with Hb to serve as oxygen carrier and demonstrated their excellent capacity of serving as microscaffolds for cell adhesion and achieved regeneration promotion of abdominal wall defect (Figure 17e,f).^[^ ^231^ ^]^

### 4.4 Other Applications

Other advanced applications involve micromotors/nanomotors and vaccine delivery. Erythrocytes could deform into a parachute‐like shape as they pass through the walls of blood vessels, which is a highly directional shape. Inspired by deformed erythrocytes, researchers have designed a variety of parachute‐like micromotors and nanomotors, based on radial depression, that can easily carry power fuels. The movement of motors is propelled by fuels, which makes them advantageous in biomedicine because they enhance the feasibility in developing more complex treatments. These micromotors are powered mostly by bubbles, including oxygen released by hydrogen peroxide (H_2_O_2_) catalyzed by platinum (Pt) or manganese dioxide (MnO_2_), and hydrogen released by magnesium (Mg) in acid solutions. These micromotors have been used in gastrointestinal drug delivery, detection, water treatment, and other applications.

**Figure 18:**
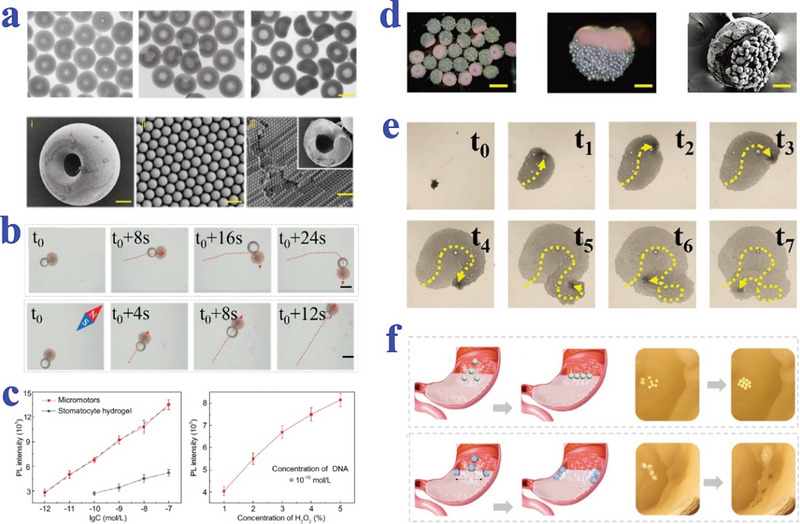
Micromotors in biomedicine. a–c) Oxygen bubbles‐driven Pt‐based micromotors are used for multiplex assays of DNA. Microscopic images and SEM images of the ELPs (a). The spontaneous movement of the micromotors (b) and their performance in multiplex detection of DNA (c). Reproduced under the terms of the CC‐BY license.^[^ ^117^ ^]^ Copyright 2019, The Authors, Published by Oxford University Press. d–f) Magnesium‐based micromotors driven by hydrogen bubbles are used for gastric delivery. Optical images and SEM image of erythrocyte‐like micromotors with adhesion capacity (d). Motion performance in simulated gastric juice (e). Adhesion actions of adhesive micromotors (the down) and traditional spherical particles (the up) in the stomach in vivo (f). Reproduced under the terms of the CC‐BY license.^[^ ^119^ ^]^ Copyright 2021, The Authors, Published by Wiley‐VCH.

Pan et al. prepared bowl‐like micromotors in which were electroless plated with Pt particles. In the H_2_O_2_ solution, Pt catalyzed the decomposition of H_2_O_2_ to generate oxygen bubbles; thus, propelling the bowl‐like micromotors to irregularly move.^[^ ^10^ ^]^ Ren et al. fabricated erythrocyte‐like micromotors with Fe_3_O_4_ and MnO_2_ NPs. Fe_3_O_4_ NPs could serve as catalysts to catalyze degradation of pollutant and guide the movement of micromotors. MnO_2_ NPs arranged on the cavity of the micromotors were utilized to propel motion in H_2_O_2_ solution by producing oxygen bubbles, which catalyzed the pollutants degradation as well. Their micromotors have performed effective catalytic performance to degrade methylene blue and subsequent easy collection by magnets. In the previous research of our group, micromotors of PhC MPs had been designed as an optical device (**Figure** **18**a–c). The micromotors with Pt and ferric oxide in their cavities achieved self‐movement, while the magnetism of ferric oxide simplified the collection process of the micromotors. The self‐movement of these micromotors could greatly accelerate the mixing efficiency of the solution samples as well as efficiently increase the interactions of targets and probes to facilitate single or multiplex detection with faster and more sensitive performance.^[^ ^117^ ^]^ Besides, the inverse opal structures of hydrogel obtained by using PhC micromotors as templates have been utilized as an ingenious drug delivery system, followed by attaching Mg particles onto the surface of hemispherical side to realize spontaneous movement (Figure 18d–f). The micromotors were driven by continual bubbles of generated hydrogen, which were products of Mg that reacted with gastric juice. Those micromotors with spontaneous movement could efficiently adhere to the region with ulcer due to their distinctive architecture in the stomach to release drugs, demonstrating great curative effect in treatment of gastric ulcer.^[^ ^119^ ^]^

As for nanomotors, varied stomatocyte nanomotors based on amphiphilic block copolymers have been reported. Wilson's group designed various stomatocyte nanomotors of PEG‐b‐PS as drug carrier candidates, which were obtained through osmotic folding of PEG‐b‐PS. The nanomotors used Pt NPs as engine to generate bubbles for movement by catalyzing decomposition of H_2_O_2_ released from cancer cells. Besides, the nanomotors exhibited excellent designability of functional design. When temperature‐sensitive polymer brush of poly(*N*‐isopropyl acrylamide) (PNIPAM) was successfully modified onto the nanomotor, the opening of the nanomotor was controlled to be enlarged or narrowed with temperature change. This was so that, movement was regulated because the access of H_2_O_2_ fuel was changed.^[^ ^88^ ^]^ When disulfide bridge was introduced to PEG‐b‐PS, the prepared nanomotors obtained redox‐responsive capacity to trigger cargo release in biological reducing environments. After the treatment with endogenous reducing agent such as GSH at a similar concentration in the cellula, the external shells of PEG could be cleaved, causing the loss of movement actions and triggering cargo release.^[^ ^97^ ^]^ When naphthalocyanine, a strong NIR light absorber, was loaded into nanomotors, the stomatocyte nanomotors performed temperature responsive on/off motion under NIR irradiation. The well‐controlled movements were based on dehydration of PEG in the PEG_44_‐b‐PS_141_ at near 55 °C, causing chain of copolymer to become hydrophobic. It was worth noting that the nanomotors could not only move propelled by H_2_O_2_ secreted from cancer cells but also ablate the tumor cells in PTT under NIR light illumination.^[^ ^91^ ^]^ After azobenzene derivatives were grafted to surfaces and the addition of b‐cyclodextrins (b‐CDs), the supramolecular nanomotors could manipulate motion and speed to adjust the access of H_2_O_2_ fuel into the nanomotors upon blue light irradiation. The speed of the nanomotors increased with the b‐CDs were detached when trans‐azobenzene moieties were isomerized to the cis‐form under blue light irradiation.^[^ ^94^ ^]^

As a better alternative, the size of nanomotors could be decreased from ≈400 nm to ≈150 nm by combining the method of extrusion. The designed ultrasmall stomatocyte nanomotors could greatly penetrate across the vasculature and enhance uptake by cancer cells.^[^ ^92^ ^]^ Besides, non‐biocompatible and non‐biodegradable glassy PS is unfortunately not ideal for further applications in biomedicine. Biodegradable PEG‐b‐PCL was introduced to stomatocyte of PEG‐b‐PS and DOX was encapsulated in the cavity of the nanomotor. The self‐assembled nanomotors could autonomously move toward tumor cells under low H_2_O_2_ concentrations. Biodegradable PCL was degraded to form large pores on the stomatocyte surface when taken up by tumor cells, leading to sustained DOX release in tumor site to kill the tumor cells.^[^ ^98^ ^]^ Another block copolymer of biodegradable PEG‐PDLLA integrating with functional azide handles was generated. Selective reduction of the stomatocyte surface provided the secondary handle for coupling fluorophores and other molecules while the inside of the stomatocyte was coupled with enzymes as engine via click‐reactions for spontaneous movement.^[^ ^99^ ^]^

Pijpers et al. also constructed biodegradable and biocompatible stomatocytes nanomotors of PEG‐PDLLA with MnO_2_ NPs in the cavity. The nanomotors could transduce chemical energy into motion and performed compatibility with cells, degradability and stability against protease deactivation. They also remained active in cellular environments and showed effective tumor penetration.^[^ ^100^ ^]^ Zhang et al. fabricated stomatocytes nanomotors of PEG‐b‐PS, which loaded IONPs inside, aiming at conveying photosensitizers of PDT to tumor tissues. The hybrid nanomotors could locate at tumor sites under magnetic field and serve as contrast agent for MRI. After being trapped by cancer cells, the nanomotor could self‐propel under propelling force of O_2_ which was decomposed from endogenous H_2_O_2_ catalyzed by IONPs. The movement of nanomotors expanded the distribution of photosensitizers for enhanced PDT efficiency.^[^ ^95^ ^]^ Zhou et al. presented a reusable and ultrastable stomatocyte nanomotor of PEG‐P(S‐co‐BMA), encapsulating Pt NPs. The utilization of BMA as a photo‐crosslinker endowed the nanomotors with great stability to withstand the corrosion of organic solvents, strong alkalis, and acids. The resultant nanomotors expanded potential application in completing delicate and complicated assignments in harsh environments.^[^ ^101^ ^]^

Another one of the main applications of EM‐NVs involves potent vaccination in immunization processes.^[^ ^232^, ^233^ ^]^ They could be utilized to immunize against specific virulence factors of bacterial toxins and the immunotherapeutic treatment of cancer.^[^ ^153^ ^]^ EM‐NVS‐NPs are shown to sequestrate bacterial toxins so that the toxins lose their motional freedom but are non‐denatured, promising safe and effective delivery of vaccine in vivo.^[^ ^234^ ^]^ In the case of staphylococcus aureus, Zhang et al. demonstrated that toxin (Hl*α* as a model) was successfully adhered to EM‐NVs‐PLGA NPs and detained; thus, inactivating the virulence mechanism of Hl*α* and inducing stronger immune response without generating any anti‐nanoparticle immune response and any immunization against the EM‐NVs. This hybrid system allowed the immune system to specifically deal with high anti‐toxin responses of nanotoxoid cargoes.^[^ ^235^ ^]^ Furthermore, this immunization strategy could be extended to vaccination of other bacteria so that the EM‐NVs‐NPs could detain other toxins of membrane‐active proteins, such as PFTs, specific small peptide in bee venom, and the oligomerizing streptolysin‐O secreted by streptococcus bacteria.

EM‐NVS‐NPs, used as vaccine formulations, have also been investigated in immunotherapy of tumors. They could present antigens to target antigen presenting cells (APCs), especially dendritic cells (DCs), and induce a cell response of antigen specific T cell. The integration of erythrocyte components would endow carrier systems with specific antigens to circulate in the blood and facilitate their target delivery to the DCs, enhancing antigen presenting efficiency and initiating a response against the tumor. Guo et al. developed EM‐NVs‐PLGA NPs as antineoplastic vaccine loading with toll‐like receptor 4 agonist, antigenic peptide (hgp10025‐33), monophosphoryl lipid, and so on. Mannose was inserted into membrane structure to actively target APCs in the lymphatic organs while redox‐sensitive peptide was conjugated to PLGA NPs to allow cleavage in the intracellular milieu. After intradermal injection, it was observed that the antigens deposited in the administration site and the retention in lymph nodes was promoted. The vaccine delivery system exhibited extended the tumor‐occurring time in prophylactic, reduced tumor metastasis in metastatic melanoma models, and restrained tumor growth in treatment.^[^ ^236^ ^]^ This research would offer a superior strategy to propose a low‐cost, effective, and safe formulation of biomimetic vaccine in tumor prevention and treatment; thus, exhibiting a novel strategy for potential cancer treatment of prophylactic and therapeutic.

## 5 Summary and Outlook

In conclusion, we have summarized the erythrocytes‐derived and erythrocytes‐inspired biomaterials, covering engineered erythrocytes, erythrocyte membrane derivatives, self‐marker modified particles, HBOCs, as well as synthesized non‐spherical ELPs that mimic the pivotal structural features of biconcave discoid morphology on biomimicry. Starting with modification of erythrocytes and fabrication strategies of erythrocytes derivatives, the applications of employing erythrocytes and EM‐NVs as drug carriers or camouflage coating of NPs to deliver therapeutic and diagnostic agent were introduced. Subsequently, self‐markers of membrane proteins and Hb were further extracted to develop stealth biomaterials of drugs carrier for administration and HBOCs for tissue regeneration in vivo, respectively. Besides, the erythrocytes morphology‐inspired particles were summarized from preparation strategies to their practical and potential applications in drug delivery, tissue engineering, and micromotors. Although with promising prospects, these systems are still faced with many limitations and there is still much room to improve.

The first issue concerns the safe, large‐scale, and affordable obtaining of erythrocytes derivatives as well as their modification and stabilization to meet the requirements of clinically relevant cell numbers. The direct use of erythrocytes faces possible infectious problems and blood‐type‐matched limitation. Membrane‐derived vesicles, recognized as the membrane fragment in nano‐size, face the same problems of erythrocytes and other new problems such as vesicle aggregation and not identical protein components from partial membrane. Besides, the preparation of erythrocytes derivatives faces expensive costs and complicated procedures compared to traditional pharmaceutical forms. All of them also lack the specificity to locate lesion for a sufficient time. Given the current findings, additional functional components need to be integrated to erythrocytes derivatives to make them specific, stable, and generally applicable. The strategies of fusing with other cell (e.g., cancer cell, immune cell) membranes, modified with target ligands, and integrated with functional polymer could be further developed to enhance their target capacity and stability in drug delivery, bioimaging, cancer therapy, and vaccine delivery in vivo. Particularly, employing hybrid membrane vesicles of erythrocyte and immune cell, cancer cell, and even bacterial as coating components of NPs would be further investigated and utilized in the future.

The second issue focuses on how to solve the drawbacks of self‐marker membrane proteins modified particles. Extracting specific membrane proteins to modify vectors instead of using erythrocyte membranes also provides a new purpose. However, there are limited kinds of membrane proteins on erythrocyte membrane which have been used as functional markers to modify biomaterials to mimic physiological activities of erythrocytes. It's worth noting that partial membrane proteins and their analogues are observed to be reactive across different species (e.g., CD59 and DAF), implying great risks to serve as self‐markers. Purification and identification bring great complexity and high cost for therapy as well. The focus of research should be on the purification and function identification of more membrane proteins, improving the efficiency of membrane protein modification and keeping functional integrity. Artificial analogues provide available alternatives of membrane proteins that are worth investigating and expecting as well.

The third issue is in regard to broadening applications of HBOCs, while reducing the adverse effects of Hb. HBOCs without toxic effects are still anticipated worldwide and attract great attentions. Exploration on combination of lipid encapsulation, polymerization, and modification into polymer particles may achieve universal application and better results in both intravenous injection and tissue regeneration. The combined strategies are more promising to create high quality HBOCs with extended shelf‐life and excellent oxygen binding affinity under convenient storage conditions. Besides, the application potentials of promising ELPs as erythrocyte substitutes still need to be explored in vivo, and availability as oxygen delivery module in scaffold and cell culture systems should advance the progress in animal tests and cell tests.

In addition, the synthetic particles mimicking erythrocytes deserve more consideration because they may combine the advantages of synthetic materials and biological entities. These functional particles with different morphology possess varied application prospects in therapeutic delivery, diagnostic, tissue engineering, and other fields. Although promising, they are still in their infancy with obstacles to overcome in their synthesis and comprehensive characterization. With regard to these functional particles, there are few methods to prepare non‐spherical particles and most of the current researches have only studied the synthesis methods. Further exploration of their practical potential in the biomedical field is lacking but worthwhile. The novel MPs/NPs are worth exploring to seek a synthetic particle that could most resemble the erythrocytes and enhance the pharmacokinetics of loaded drugs, and pursue physiological characteristics and complexity of erythrocytes. Molecules encapsulated in those systems could also be responsively released upon external stimuli when stimuli‐responsive biomaterials are integrated into the substrate material of ELPs, presenting novel and promising opportunities for the vectorization delivery of active molecules.

## Conflict of Interest

The authors declare no conflict of interest.
